# Mild Hyperthermia Accelerates Bone Repair by Dynamically Regulating iNOS/Arg1 Balance in the Early Stage

**DOI:** 10.1002/advs.202409882

**Published:** 2024-12-31

**Authors:** Jinhui Zhao, Yiping Luo, Lei Zhang, Yunfeng Chen, Yixing Chen, Xinhui Wu, Aihemaitijiang Aierken, Dilixiati Duolikun, Tianlong Wang, Zifei Zhou, Zhiqing Liu, Longpo Zheng

**Affiliations:** ^1^ Department of Orthopedics Shanghai Tenth People's Hospital, School of Medicine Tongji University Shanghai 200072 China; ^2^ Shanghai Trauma Emergency Center Shanghai 200072 China; ^3^ Orthopedic Intelligent Minimally Invasive Diagnosis & Treatment Center Shanghai Tenth People's Hospital Tongji University School of Medicine Shanghai 200072 China

**Keywords:** bone repair, iNOS/Arg1 balance, macrophage, mild hyperthermia

## Abstract

Mild hyperthermia therapy has garnered interest as an adjunctive treatment for bone repair. However, its optimal timing, duration, and underlying mechanisms remain unclear. In this study, how mild hyperthermia supports bone repair during the early stages is assesed. These findings reveal that mild hyperthermia accelerates bone regeneration by dynamically regulating inducible nitric oxide synthase/arginase 1 (iNOS/Arg1) balance. This process involves macrophage polarization to the M1 phenotype through *iNOS* activation, followed by a rapid transition to the M2 phenotype through *Arg1* activation after 3 days of sustained mild hyperthermia. RNA‐Seq reveals that a single day of mild hyperthermia induced immune alterations aligned with the early inflammatory phase of bone repair, characterized by osteoclast activation, cell recruitment, and neovascularization, thereby preparing for the transition to the repair phase. Experiments involving subcutaneous abscesses, subcutaneous embedding, and critical cranial bone defects further confirm that early mild hyperthermia treatment dynamically regulates macrophage phenotypes. This regulation enhances early antibacterial activity, promotes angiogenesis, and facilitates the transition from inflammation to repair, ultimately accelerating bone‐defect repair. This study is the first to elucidate the dual temporal effects of early mild hyperthermia on immune regulation, offering insights into the optimal timing and duration of photothermal therapy following bone repair surgery.

## Graphical abstract

Early and sustained photothermal therapy exerts a dual immunoregulatory effect on macrophages. This effect is mediated by near‐infrared‐induced mild hyperthermia, which dynamically adjusts the iNOS/Arg1 balance through L‐arginine metabolism. By modulating macrophage polarization, this approach accelerates the shift from an inflammatory to a reparative state, thereby creating a favorable environment that promotes faster bone regeneration and enhances overall healing efficiency.

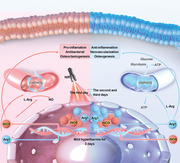

## 1 Introduction

Critical‐sized bone defects caused by tumor resection, traumatic fractures, or aseptic necrosis remain a significant global health concern and challenge for orthopedic surgeons.^[^ ^1^ ^]^ Because of the limited availability of autologous bone grafts and the potential risks associated with allogeneic bone grafts,^[^ ^2^ ^]^ repairing bone defects exceeding a critical size often requires bone biomaterials.^[^ ^3^ ^]^ The decellularized extracellular matrix (dECM) is a structured assembly of proteins and polysaccharides synthesized by cells, creating a physical environment in which cells reside. It exhibits exceptional biocompatibility and is a fundamental feature of multicellular organisms, providing structural support and organization for cells within tissues. This has garnered significant attention and spurred extensive exploration of various applications.^[^ ^4^ ^]^

In recent years, biophysical stimulation has garnered increasing attention as a noninvasive, easy‐to‐operate, and controllable therapeutic tool, specifically in patients with postoperative rehabilitation of bone fractures. This approach complements the use of bone implants^[^ ^5^ ^]^ and has been integrated into non‐pharmacological treatment in traditional Chinese medicine for centuries, supported by extensive clinical practice and experience. Methods, such as mechanical stimulation through long‐term traction and acupuncture, mild hyperthermia stimulation with moxibustion and fumigation, and negative pressure techniques (cupping therapy) indicate the significance of biophysical stimulation on physiological functions and cellular metabolism. These traditional practices highlight the significance of physical stimulation in alleviating disease symptoms and facilitating the recovery of bodily functions. Currently, biophysical stimulation is extensively applied in bone repair. Among various modalities, thermal stimulation is a straightforward and effective method. This aligns with the physiological processes involved in fracture repair.

Following a fracture, a hematoma forms around the fracture site owing to trauma and tissue damage caused by the broken bone ends. This process involves blood outflow within the cancellous bone at the fracture site and rupture and bleeding of the surrounding capillaries. Because of the abundant blood flow and inflammatory cascade, the hematoma localized around the fracture exhibits a slightly elevated tissue temperature, establishing an injury repair hyperthermic microenvironment, which plays a crucial role in tissue repair and the body's response to traumatic factors. The elevated temperature enhances immune cell activity, aiding in nonspecific immune responses to prevent infection risks in later stages and significantly facilitating neovascularization. Neovascularization is crucial for recruiting, proliferating, and differentiating stem cells during fracture repair.^[^ ^6^ ^]^ Clinical evidence indicates that, in thermostable animals, body temperature rises by only 1–2 °C during fever, with localized increases in skin temperature observed around the fracture. These findings indicate that mild hyperthermia stimulation can regulate cell growth, enhance metabolic activity, and facilitate bone tissue repair.^[^ ^7^ ^]^ Thermal stimulation from an external heat source (2–4 °C above body temperature) significantly enhances the osteogenic activity of stem cells, including the expression of alkaline phosphate (ALP) and the deposition of calcium nodules.^[^ ^8^ ^]^ Thermal stimulation induces the upregulation of heat shock proteins (HSPs), which activate the extracellular signal‐regulated kinase pathway, thereby facilitating osteogenic differentiation.^[^ ^9^ ^]^ The relationship between temperature and mineralization demonstrated that ALP activity increases within a specific temperature range. Based on these insights, photothermal therapy has been introduced for the treatment of bone defects.^[^ ^10^ ^]^

**Scheme 1:**
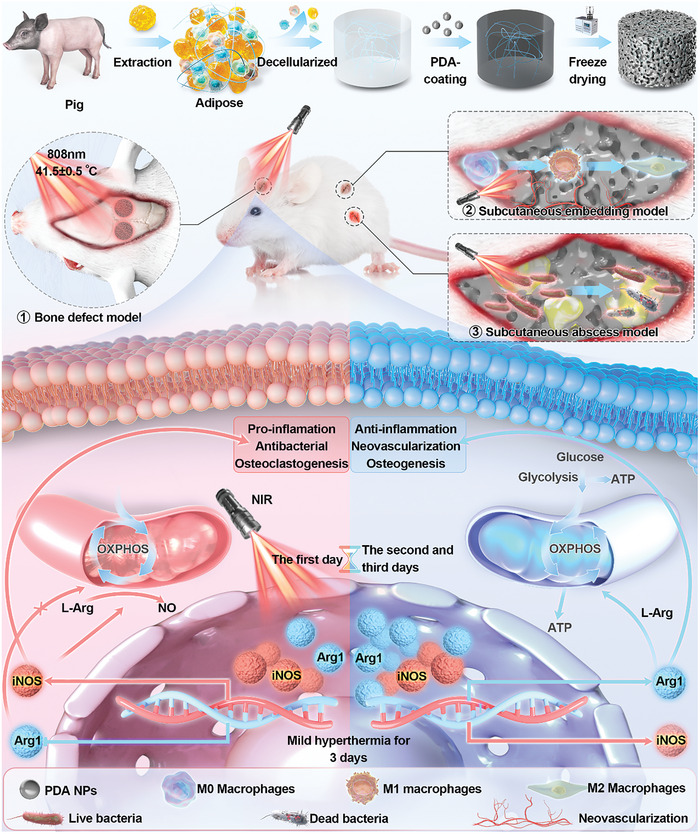
Synthesis of p‐DAT+NIR is schematically illustrated, highlighting the potential mechanism that mild hyperthermia accelerated bone repair by regulating iNOS/ Arg1 balance in the early stage.

Current research on the effects of mild hyperthermia on bone repair has primarily focused on assessing its beneficial effects on osteogenic differentiation. However, recent advances in osteoimmunology have highlighted the significance of the immune system in osseointegration.^[^ ^11^ ^]^ The prevailing view in the existing literature on mild hyperthermia‐mediated immune regulation suggests that mild hyperthermia can shift the pro‐inflammatory phenotype to an anti‐inflammatory/repair phenotype, primarily in cases of inflammatory or pathological bone defects. However, how mild hyperthermia regulates the immune response in ordinary critical‐sized bone defects remains unclear.^[^ ^12^ ^]^ Additionally, the optimal timing, duration, and underlying mechanisms of this therapy for facilitating bone regeneration remain unclear. In this study, we comprehensively assessed the biosafety and recruitment effects of mild hyperthermia on bone repair‐associated stem cells. Moreover, we assessed the effects of sustained mild hyperthermia on polarized macrophages during the early stages of bone repair. Interestingly, we observed that mild hyperthermia facilitated the shift of macrophages to the M1 phenotype on the initial day of treatment, aligning with the inflammatory phase of bone repair. Subsequent mild hyperthermia treatment further facilitated the transition of macrophages to the repair phenotype by day 3 by dynamically regulating the inducible nitric oxide synthase/arginase 1 (iNOS/Arg1) balance (**Scheme** **1**). This mechanism significantly accelerated the transition from the inflammatory phase to the repair phase. This study offers a theoretical foundation for postoperative rehabilitation following bone repair and has significant clinical relevance in practical applications.

## 2 Results and Discussion

### 2.1 Biocompatibility of Mild Hyperthermia

**Figure 1:**
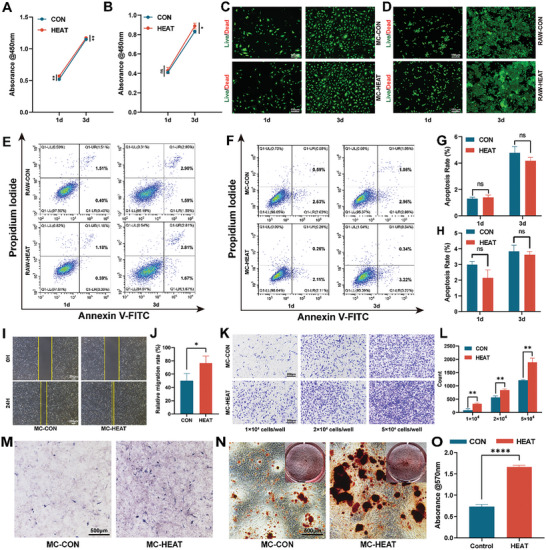
The effects of mild hyperthermia on cell viability, migration, and osteogenic differentiation. A) CCK‐8 results of RAW cells cultured under mild hyperthermia for 1 day and 3 days (n = 3). B) CCK‐8 results of MC‐14 cells cultured under mild hyperthermia for 1 day and 3 days (n = 3). C) Live/Dead staining of MC‐14 cells cultured under mild hyperthermia for 1 day and 3 days. D) Live/Dead staining of RAW cells cultured under mild hyperthermia for 1 day and 3 days. E) Apoptosis flow cytometry of RAW cells cultured under mild hyperthermia for 1 day and 3 days. F) Apoptosis flow cytometry of MC‐14 cells cultured under mild hyperthermia for 1 day and 3 days. G) Flow cytometry quantitative analysis of RAW cells (n = 3). H) Flow cytometry quantitative analysis of MC‐14 cells (n = 3). I) Scratch assay of MC‐14 cells. J) Quantitative analysis of the scratch assay (n = 3). K) Transwell experiments with MC‐14 cells at different concentrations. L) Quantitative analysis of the Transwell experiment (n = 3). M) ALP staining of mild hyperthermia‐stimulated osteogenesis. N) ARS staining of mild hyperthermia‐stimulated osteogenesis. O) Quantitative analysis of ARS staining (n = 3). (^*^ *p* < 0.05, ^**^ *p* < 0.01, ^****^ *p* < 0.0001).

In this study, cells were incubated at 41.5 °C to simulate a mild hyperthermia therapy microenvironment and exposed to this condition for 15 min daily. The Cell Counting Kit‐8 (CCK‐8) assay demonstrated that the proliferation of RAW cells after 1 and 3 days of mild hyperthermia treatment was significantly higher than that of the control (CON) group (**Figure** **1**A). For MC‐14 cells, mild hyperthermia stimulation for 1 day had no significant effect on cell proliferation. However, after 3 days of continuous mild hyperthermia stimulation, cell proliferation in the experimental group was significantly higher than that in the control group (Figure 1B). The above results support previous studies that mild hyperthermia can facilitate cell proliferation.^[^ ^13^ ^]^ Additionally, live/dead cell staining, apoptosis flow cytometry analysis, and quantitative results demonstrated that mild hyperthermia did not affect cell viability (Figure 1C–H). There was no significant abnormal cell death in either the CON or hyperthermia exposure and treatment (HEAT) group. This indicates that mild hyperthermia is a highly biocompatible adjunctive therapy.

### 2.2 Mild Hyperthermia Facilitates Cell Recruitment

The recruitment of seed cells is crucial for tissue engineering and repair. The rapid and early recruitment of these cells, which serve as raw materials for tissue repair, aids in accelerating bone regeneration. Osteoblasts, crucial for bone defect repair, were assessed for their recruitment under mild hyperthermic conditions. Both scratch and Transwell assays demonstrated that the HEAT group exhibited a significantly stronger ability to facilitate cell migration compared to that of the CON group (Figure 1I–L). These results indicate that mild hyperthermia treatment can significantly enhance the migration of osteoblasts, thereby facilitating the repair of bone defects.

### 2.3 Mild Hyperthermia Facilitates Cell Osteogenesis

ALP is a marker of early‐stage osteogenic differentiation. Alizarin Red staining (ARS) reflects calcium nodule formation, indicating later‐stage osteogenic activity.^[^ ^14^ ^]^ In this study, the mildly controlled (MC)‐HEAT group exhibited enhanced ALP activity after 7 days of osteogenic induction, which included mild hyperthermia stimulation for the initial 3 days (Figure 1M; Figure S1, Supporting Information). ARS staining and quantitative analysis revealed that the MC‐HEAT group generated more calcium nodules than that of the MC‐CON group (Figure 1N,O). Mild hyperthermia at 41–42 °C can enhance the expression of downstream osteogenesis‐related proteins, such as Runt‐related transcription factor 2 and type I collagen, by activating the alkaline phosphatase/phosphorylated protein kinase B (AKP/pAKT)/HSP90 signaling pathway, thereby facilitating the osteogenic activity of osteoblasts.^[^ ^12^, ^13^ ^]^ Additionally, our previous studies on mild magnetic hyperthermia have demonstrated that alternating magnetic fields that penetrate tissues effectively can induce mild hyperthermia in composites (41–42 °C). This mild hyperthermia significantly facilitates the osteogenic differentiation and biomineralization of pre‐osteoblasts through the HSP90‐activated PI3K/AKT pathway.^[^ ^15^ ^]^ In this study, we observed that applying mild hyperthermia using an incubator facilitates osteogenic differentiation.

### 2.4 Mild Hyperthermia Modulates Macrophages

To date, research in this field has predominantly focused on enhancing the osteogenic induction of osteoblasts through diverse methods.^[^ ^16^ ^]^ However, despite promising in vitro results, certain bone implant materials have exhibited reduced efficacy in vivo.^[^ ^17^ ^]^ This discrepancy stems from the complex cascade of reactions involved in foreign‐material‐mediated bone repair. These reactions primarily encompass four interrelated and synergistic phases: hematoma formation and inflammatory response, angiogenesis, novel bone formation, and bone remodeling,^[^ ^18^ ^]^ which are heavily influenced by macrophages.^[^ ^19^ ^]^

**Figure 2:**
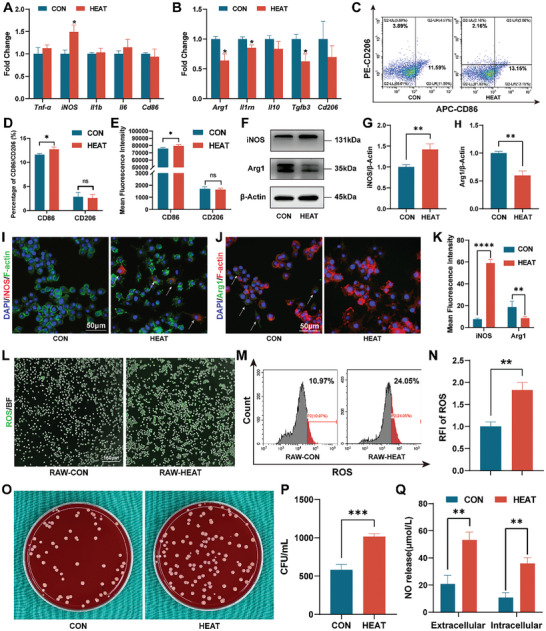
Regulation of macrophages by mild hyperthermia. A) mRNA expression of M1‐macrophage‐related genes (n = 3). B) mRNA expression of M2‐macrophage‐related genes (n = 3). C) Flow cytometry of CD86 and CD206‐based FC. D) Quantitative analysis of the percentages of CD86 and CD206‐based FC (n = 3). E) Mean fluorescence intensity of CD86 and CD206 (n = 3). F) Results of Western blot for iNOS and Arg1. G) Quantitative analysis of iNOS grayscale values (n = 3). H) Quantitative analysis of Arg1 grayscale values (n = 3). I) Immunofluorescent staining of iNOS (white arrows). J) Immunofluorescent staining of Arg1 (white arrows). K) Quantitative analysis of the mean fluorescence intensity of iNOS and Arg1‐based IF (n = 3). L) ROS fluorescent staining following 1 day of mild hyperthermia treatment. M) Flow cytometry for ROS. N) Quantitative analysis of the mean fluorescence intensity of ROS‐based FC (n = 3). O) Phagocytosis assay of macrophages using bacteria‐coated coverslips. P) Quantitative analysis of bacterial colony counts (n = 3). Q) Intracellular and extracellular NO concentrations (n = 3). (^*^ *p* < 0.05, ^**^ *p* < 0.01, ^***^ *p* < 0.001, ^****^ *p* < 0.0001).

To assess the effect of mild hyperthermia on early immune regulation during bone repair, we assessed phenotypic alterations in macrophages after 1 day of mild hyperthermia. In comparison to the CON group, macrophages in the HEAT group exhibited significantly elevated levels of iNOS after 1 day of mild hyperthermia (**Figure** **2**A). However, other M1‐related markers, such as *Tnf‐α*, *Il1b*, *Il6*, and *Cd86* did not exhibit significant differences, indicating that early mild hyperthermia can facilitate the inflammatory phenotype of macrophages specifically through *iNOS* upregulation. Regarding the regulation of M2 macrophages, the HEAT group significantly reduced the expression of *Arg1*, *Il1rn*, and *Tgfb3*. Other M2‐related genes, such as *Il10* and *Cd206*, exhibited reduced expression but did not exhibit significant differences (Figure 2B). Considering the overall M1‐ and M2‐related gene indicators, early mild hyperthermia exerts its biological effects by upregulating M1‐related genes and downregulating M2‐related genes, aligning with the inflammatory phase of early bone repair.^[^ ^20^ ^]^

Subsequently, we used flow cytometry (FC) to assess the macrophage surface markers following mild hyperthermia. Antigen‐presenting cell (APC)‐labeled CD86 was used to identify M1 macrophages, whereas phycoerythrin (PE)‐labeled CD206 was used to label M2 macrophages. Consistent with the reverse transcription‐quantitative polymerase chain reaction (RT‐qPCR) findings, the HEAT group demonstrated increased surface expression of CD86 and reduced expression of CD206 on macrophages (Figure 2C). Quantitative analysis demonstrated significantly higher proportions and fluorescence intensity of CD86 in the HEAT group than in the CON group. Although the proportion and fluorescence intensity of CD206 were lower in the HEAT group, these differences were not statistically significant, aligning with the qPCR analysis (Figure 2D,E). Western blot (WB) analysis indicated increased *iNOS* expression and reduced *Arg1* expression in the HEAT group (Figure 2F–H). We conducted immunofluorescence experiments for iNOS and Arg1, demonstrating that the fluorescence intensity of iNOS was significantly higher in the HEAT group, whereas that of Arg1 was significantly lower than that in the CON group (Figure 2I–K). iNOS and Arg1 are crucial enzymes in inflammation—iNOS facilitates pro‐inflammatory responses, and Arg1 facilitates anti‐inflammatory responses by catalyzing L‐Arg, thereby serving dual functions.^[^ ^21^ ^]^ Additionally, the transcriptional level of Arg1 can affect energy metabolism by regulating the mitochondrial oxidative respiratory chain, thereby affecting bone homeostasis.^[^ ^22^ ^]^

Amino acids and their metabolism are vital to macrophage immunity.^[^ ^23^ ^]^ Early inflammation raises mitochondrial reactive oxygen species (ROS), driving M1 polarization. In this process, arginine is converted to citrulline and nitric oxide (NO) by iNOS, inhibiting mitochondrial complexes I and II and compromising integrity.^[^ ^23a^ ^]^ In contrast, M2 macrophages sustain a robust tricarboxylic acid (TCA) cycle and enhanced oxidative phosphorylation (OXPHOS). A defining feature of M2 macrophages is Arg1, which converts arginine to ornithine for polyamine synthesis. Intracellular arginine can also originate from phagocytosed apoptotic cells. The metabolite putrescine stabilizes Mcf2 mRNA, activating Rac1 and enhancing apoptotic body uptake. Additionally, spermidine derived from arginine promotes the expression of proteins involved in the TCA cycle and OXPHOS.^[^ ^23a^ ^]^

The fluorescent images of ROS stained by 2′7’‐dichlorodihydrofluorescein diacetate (DCFH‐DA) illustrated that the fluorescence intensity in the RAW‐HEAT group was significantly higher than that in the RAW‐CON group (Figure 2L; Figure S2, Supporting Information). This indicates that mild hyperthermia may enhance ROS production by the macrophages. Flow cytometry revealed that 10.97% and 24.05% of cells in the RAW‐CON and RAW‐HEAT groups were fluorescein isothiocyanate (FITC)‐ROS positive, respectively (Figure 2M,N). This percentage is significantly higher than that observed in the RAW‐CON group and aligns with the findings of previous studies on mild magnetic hyperthermia.^[^ ^24^ ^]^ Increased ROS levels are associated with the macrophage phenotype, with excessive ROS potentially polarizing macrophages to the M1 phenotype. In contrast, M1 macrophages can further increase ROS production.^[^ ^25^ ^]^ In this study, we observed that early mild hyperthermia significantly upregulated the iNOS phenotype. iNOS catalyzes the production of NO from L‐Arg, thereby contributing to the synthesis of ROS and antimicrobial agents.^[^ ^21^ ^]^ In this study, the mild hyperthermia‐facilitated antimicrobial effects primarily stem from two aspects. First, iNOS activation enhances macrophage phagocytic activity, thereby increasing their capacity to engulf bacteria. Therefore, the HEAT group exhibited significantly higher intracellular bacterial counts than those of the CON group (Figure 2O,P). Second, iNOS catalyzes increased NO production from L‐Arg (Figure 2Q), which can directly eliminate bacteria through its antimicrobial action (Figure S3, Supporting Information).

### 2.5 The Transcriptomic Profile of Macrophages Stimulated by Mild Hyperthermia

**Figure 3:**
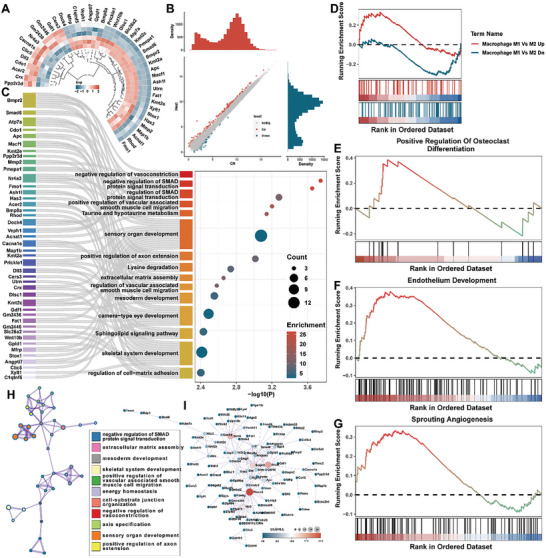
Exploration of gene expression patterns and functional enrichment analysis. A) Circular heatmap. B) Scatter plot. C) Biological processes enriched by differentially expressed genes. D) GSEA analysis of macrophage polarization‐related genes. E) GSEA analysis of osteoclast differentiation‐related genes. F) GSEA analysis of endothelial development‐related genes. G) GSEA analysis of angiogenesis‐related genes. H) Metascape enrichment network visualization. I) Protein‐protein interaction network of differentially expressed genes.

Transcriptomic sequencing analysis was performed on macrophages after 1 day of mild hyperthermia. Differential analysis identified a total of 223 differentially expressed genes (164 upregulated and 59 downregulated). Circular heatmap (**Figure** **3**A) and scatter plot (Figure 3B) illustrate differences in gene expression. For the biological processes involved in the gene groups affected through heat stimulation in macrophages, the clusterProfiler package was used for enrichment analysis of differentially expressed genes (DEGs) (*p* < 0.05). Figure 3C illustrates the DEG‐enriched biological processes, highlighting a significant correlation between heat stimulation and selected biological processes. Gene Set Enrichment Analysis (GSEA) was used to analyze the correlation of specific biological processes. GSEA demonstrated that heat stimulation in macrophages upregulated processes associated with M1 macrophage regulation (Figure 3D) and was associated with the positive regulation of osteoclast differentiation (Figure 3E), endothelial development (Figure 3F), and angiogenesis (Figure 3G). Interestingly, Metascape enrichment network visualization revealed the sharing of genes across enriched pathways, with greater gene overlap observed in pathways related to axis specification, sensory organ development, and the positive regulation of axon extension. Additionally, gene sharing was also noted in processes such as extracellular matrix assembly, skeletal system development, and the positive regulation of vascular‐associated smooth muscle cell migration, suggesting that mild hyperthermia is closely linked to these biological processes (Figure 3H). Furthermore, the protein‐protein interaction network illustrated the mechanisms of protein interactions among DEGs affected by mild hyperthermia. The connectivity with potentially relevant interacting proteins indicates that mild hyperthermia may exert its effects through key genes such as Hmecn2, Rhod, Bmpr2, Birc6, and Me3 (Figure 3I).

Analysis of the sequencing results revealed that 1 day of mild hyperthermia facilitated endothelial cell proliferation (Figure S4, Supporting Information), migration (Figure S5, Supporting Information), and blood vessel morphogenesis (Figure S6, Supporting Information). This aligns with our in vitro findings and indicates that early‐stage mild hyperthermia is closely associated with angiogenesis. Additionally, GSEA demonstrated that mild hyperthermia stimulation can inhibit apoptosis (Figure S7, Supporting Information), further confirming the safety of this treatment. Early mild hyperthermia stimulated osteoclast activity and positively influenced ossification (Figure S8, Supporting Information). This indicates that early mild hyperthermia can enhance bone metabolism by increasing the activities of both osteoblasts and osteoclasts. In the early stages of bone repair, this dual effect helps to rapidly clear necrotic bone and accelerate the overall repair process. Additionally, we observed that after mild hyperthermia treatment, macrophages exhibited increased responsiveness to nitrogen compounds (Figure S9, Supporting Information), which may correlate with our earlier finding that mild hyperthermia facilitates NO release.

### 2.6 The Effect of Immune Regulation on Osteogenesis

**Figure 4:**
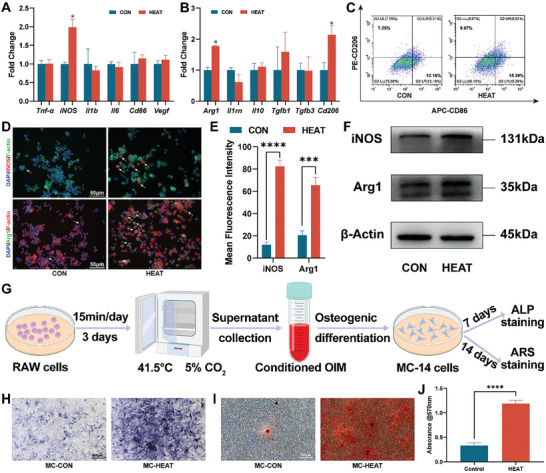
The impact of immune regulation on osteogenesis. A) mRNA expression of M1‐macrophage‐related genes (n = 3). B) mRNA expression of M2‐macrophage‐related genes (n = 3). C) Flow cytometry of CD86 and CD206‐based FC. D) Immunofluorescent staining of iNOS (white arrows) and Arg1 (white arrows). E) Quantitative analysis of the mean fluorescence intensity of iNOS and Arg1‐based IF (n = 3). F) Results of Western blot for iNOS and Arg1. G) Schematic diagram of osteogenic induction conditioned medium configured with macrophage supernatant to promote osteogenic differentiation of MC‐14 cells. H) ALP staining cocultured with immunoregulatory RAW cells. I) ARS staining cocultured with immunoregulatory RAW cells. J) Quantitative analysis of ARS staining (n = 3). (^*^ *p* < 0.05, ^***^ *p* < 0.001, ^****^ *p* < 0.0001).

An acute inflammatory response is initiated within the first day following bone injury.^[^ ^26^ ^]^ Excessive or prolonged inflammation can hinder bone repair,^[^ ^27^ ^]^ highlighting the need for a transition from the inflammatory phase to the repair phase. We exposed macrophages to mild hyperthermia for 15 min daily for 3 days and subsequently assessed their effects on the cells. The mRNA results for the M1 phenotype were similar to those observed after 1 day of treatment, with only the *iNOS* gene being significantly upregulated (**Figure** **4**A). However, the M2‐related mRNA results differed significantly from those after 1 day of treatment. All genes except *Il1rn* exhibited an upward pattern, with significantly higher expression levels of *Arg1* and *Cd206* in the HEAT group than those in the CON group (Figure 4B). Early mild hyperthermia stimulation induces the production of HSP70,^[^ ^28^ ^]^ which is released from target cells into the local microenvironment. In this context, HSP70 activates macrophages via danger‐associated molecular patterns (DAMPs), leading to the release of substantial quantities of pro‐inflammatory factors such as TNF‐α, IL1β, IL6, and IL12, thereby promoting macrophage polarization toward the M1 phenotype.^[^ ^29^ ^]^ Subsequently, sustained mild hyperthermia further accelerates the transition of M1 macrophages to M2 macrophages.^[^ ^30^ ^]^ The acute inflammatory phase of bone repair occurs within the first week following a fracture, with inflammation peaking on the first day.^[^ ^31^ ^]^ The sustained high expression of *iNOS* aligns with the physiological phase of bone repair, whereas the mild hyperthermia treatment rapidly transitions macrophages from a pro‐inflammatory to a repair phenotype, facilitating a rapid postoperative bone recovery. Similarly, FC results indicated that, compared to the CON group, the proportions of CD86 and CD206 were significantly increased in the HEAT group (Figure 4C). Subsequently, we conducted immunofluorescence experiments for iNOS and Arg1. Consistent with the RT‐qPCR results, the immunofluorescence intensities of both iNOS and Arg1 were significantly higher in the HEAT group than in the CON group (Figure 4D,E). Additionally, WB experiments for the characteristic proteins iNOS and Arg1 confirmed that macrophages subjected to mild hyperthermia for 3 days exhibited high expression levels of both proteins (Figure 4F). ROS staining of RAW cells following 3 days of mild hyperthermia treatment showed that the fluorescence intensity in the RAW‐HEAT group was significantly lower than that in the RAW‐CON group (Figure S10, Supporting Information). This indicates that a continuous mild hyperthermia treatment for 3 days can accelerate the transition of M1 macrophages to M2 macrophages.

To assess the effect of macrophage immune phenotypes on osteogenic differentiation, we collected the supernatant from macrophages subjected to mild hyperthermia stimulation for 1 day or 3 days. This was used to prepare an osteogenic induction medium, which was subsequently co‐cultured with MC‐14 cells. ALP and ARS staining was performed after 7 and 14 days of culture, respectively (Figure S11, Supporting Information; Figure 4G). The ALP and ARS staining areas of MC‐14 cells co‐cultured with a conditioned medium from 1 day of mild hyperthermia stimulation were significantly smaller than those of the control group, indicating that the early inflammatory environment inhibits osteogenic differentiation. However, following 3 days of mild hyperthermia, the results demonstrated that an osteogenic induction medium prepared from the supernatant of mild hyperthermia‐treated macrophages resulted in MC‐14 cells exhibiting a larger area of ALP staining and increased calcium nodule formation (Figure 4H–J; Figure S12, Supporting Information). These results indicate that mild hyperthermia treatment establishes a more favorable immune microenvironment for early‐phase bone repair.

### 2.7 Characterization and Properties of p‐DAT+NIR

**Figure 5:**
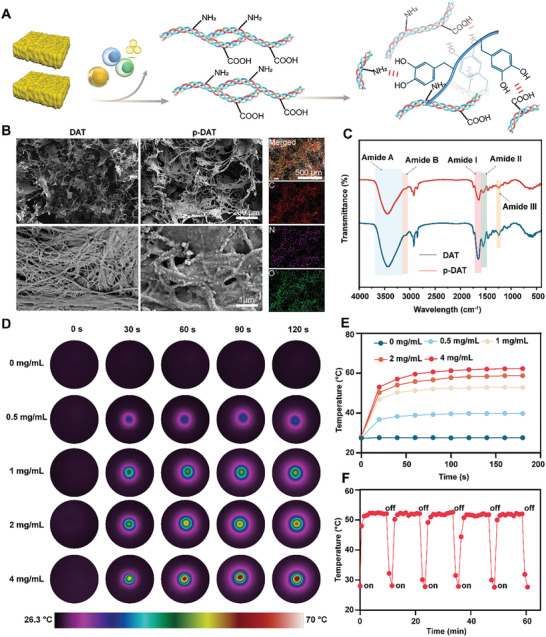
Characterization and property of p‐DAT+NIR. A) Schematic illustration of the preparation of p‐DAT scaffolds. B) SEM images and elemental mapping of fractured cross‐sections of DAT and p‐DAT scaffolds. C) FTIR spectra of DAT and p‐DAT scaffolds. D) Photothermal images of p‐DAT scaffolds with varying DA concentrations of 0, 0.5, 1, 2, and 4 mg mL^−1^. E) Photothermal curves of p‐DAT scaffolds with varying DA concentrations of 0, 0.5, 1, 2, and 4 mg mL^−1^. F) Photostability of p‐DAT scaffolds with DA concentrations of 1 mg mL^−1^.

Previous studies have assessed diverse approaches, including decellularized or devitalized alternatives in orthopedics, aimed at enhancing bone regeneration.^[^ ^32^ ^]^ Recently, decellularized adipose tissue (DAT), a form of dECM, that is readily accessible, has emerged as a viable option for repairing bone defects.^[^ ^33^ ^]^ In this study, DAT was used as a tissue engineering scaffold for bone regeneration (**Figure** **5**A). Histological staining, including hematoxylin and eosin (HE), Masson, Oil Red O, and 4′,6‐diamidino‐2‐phenylindole (DAPI) staining, demonstrated the successful extraction of DAT (Figure S13, Supporting Information). Additionally, previous studies have demonstrated that near‐infrared (NIR) light‐induced local hyperthermia accelerates bone regeneration. Phototherapy with targeted NIR light is commonly used for its precise control and minimal side effects.^[^ ^34^ ^]^

Polydopamine (PDA) is known for its robust absorbance of NIR light, which makes it an effective photothermal agent. In this study, PDA was integrated into DAT to enhance its photothermal properties. Scanning electron microscopy (SEM) analysis illustrated the porous architecture of both DAT and PDA‐modified (p‐DAT) scaffolds (Figure 5B), facilitating the optimal diffusion of nutrients and oxygen essential for tissue repair.^[^ ^35^ ^]^ Additionally, the collagen structure of the DAT was clearly observed. Following dopamine (DA) modification, the collagen surface displayed a distinct coating of the PDA layer. Elemental mapping images indicated that the p‐DAT scaffolds predominantly consisted of carbon (C), oxygen (O), and nitrogen (N) (Figure 5B; Figure S14, Supporting Information).

The Fourier transform infrared spectroscopy (FTIR) spectra of DAT and p‐DAT exhibited significant similarities, indicating a relatively thin PDA coating (Figure 5C). The FTIR spectra of DAT were aligned with those reported in a previous study.^[^ ^36^ ^]^ Figure 5C displayed distinct amide bands (amide A, amide B, amide I, amide II, and amide III) that are characteristic of collagen in both DAT and p‐DAT. To assess the stability of PDA binding on the DAT surface, we analyzed the UV–vis spectra of the supernatant solutions from p‐DAT with or without NIR (Figure S15, Supporting Information). The spectra demonstrated stable PDA binding on the DAT surface. Additionally, increasing the DA concentration resulted in a color change (white to dark brown) in both the DAT and DAT scaffolds (Figures S16 and S17, Supporting Information). To determine the optimal DA concentration, we assessed the photothermal performance of p‐DAT with varying DA concentrations under an 808 nm NIR laser at 0.75 W cm^−2^. Figure 5D illustrates that the temperature increases of p‐DAT demonstrated a concentration‐dependent pattern, reaching 27.6, 39.8, 52.8, 58.7, and 62.3 °C at DA concentrations of 0, 0.5, 1, 2, and 4 mg mL^−1^, respectively (Figure 5D,E). Additionally, the p‐DAT demonstrated excellent photostability over five on/off irradiation cycles for 60 min (Figure 5F).

### 2.8 Mild Hyperthermia Assists in the Early Inhibition of Bacteria In Vivo

Sterility is essential for surgical procedures. However, certain surgeries, such as those involving open bone defects owing to trauma or long‐duration operations on large wounds, still pose a risk of infection. If bone implant materials possess antibacterial properties, they can better prevent early infections, thereby enhancing the success of surgery and ensuring patient recovery.

**Figure 6:**
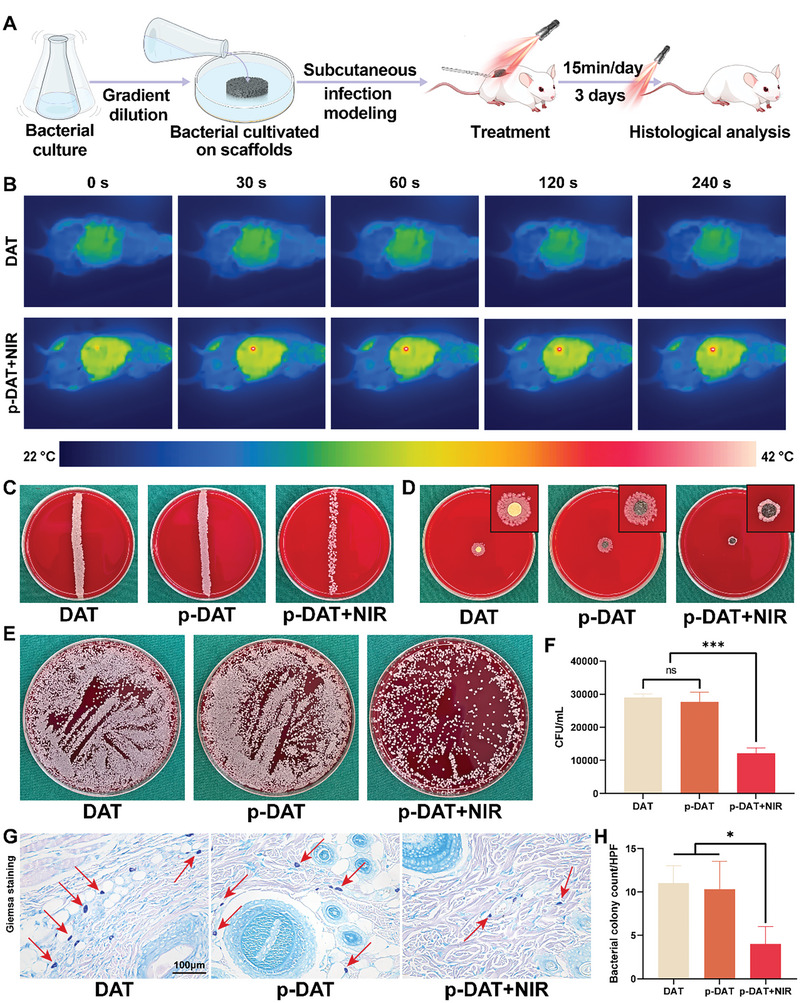
Mild hyperthermia assists in the early inhibition of bacteria. A) Schematic diagram of the subcutaneous abscess model. B) Thermal images of the DAT and DAT + NIR groups for 240s in vivo. C) The bacterial colonies of sliding cultures from explanted scaffolds. D) Colony formation experiment with scaffolds removed subcutaneously. E) spread plate method results of biofilms detached from scaffolds. F) Counting results in groups with different concentrations by spread plate method (n = 3). G) Giemsa staining images of skins from different groups (red arrows represent the clusters of stained bacteria). H) The quantitative analysis of Giemsa staining (n = 3). (^*^ *p* < 0.05, ^***^ *p* < 0.001).

In vitro, we demonstrated that macrophages stimulated through mild hyperthermia can directly phagocytose and kill bacteria and produce NO to assist in bacterial eradication. The antibacterial effect of mild hyperthermia was confirmed through in vivo experiments. **Figure** **6**A illustrates the construction flowchart of an in vivo antibacterial animal model. In summary, the bacterial solution was diluted to the appropriate concentration, and the scaffold was immersed in this solution for 6–8 h. Subsequently, a skin pocket model was created on the back of a rat, and the bacterial solution‐soaked scaffold was implanted subcutaneously. After suturing, the first phototherapy session was performed, followed by continuous illumination for 3 days (15 min per day) before conducting the histological assessments. The in vivo photothermal imaging results indicate that under NIR irradiation, p‐DAT can reach ≈40 °C within ≈30 s. By adjusting the intensity, we maintained the temperature between 40 and 42 °C. In contrast, the DAT scaffold without PDA did not exhibit any temperature increase under NIR irradiation (Figure 6B).

After 3 days of mild hyperthermia treatment, the scaffolds were removed and subjected to a direct plate streaking experiment. Holding one end of the scaffold with tweezers, we streaked it from top to bottom on a blood agar plate. The results indicated that bacterial counts in the DAT and p‐DAT groups were significantly higher than those in the p‐DAT+NIR group (Figure 6C). Additionally, we performed a bacterial formation zone experiment by placing the removed scaffolds directly at the center of a blood agar plate. After 1 day, the radius of the bacterial colonies surrounding the scaffolds in the DAT and p‐DAT groups was significantly larger than that in the p‐DAT+NIR group (Figure 6D).

To quantify the bacterial colonies retained within the scaffolds, we placed the scaffolds from each group into centrifuge tubes containing phosphate‐buffered saline (PBS). we used ultrasound to disperse the bacteria from the scaffolds into the solution. Subsequently, we plated an appropriate amount of the bacterial solution for quantification. The results exhibited no significant differences in the internal bacterial counts between the DAT and p‐DAT groups, whereas the internal bacterial count in the p‐DAT+NIR group was significantly lower than that in the other two groups (Figure 6E,F). Giemsa staining was performed to assess bacterial residues in the surrounding tissues. The results (Figure 6G, red arrows) indicated that the p‐DAT+NIR group had fewer bacterial colonies than that of the DAT and p‐DAT groups. Quantitative analysis confirmed that the number of bacterial colonies in the p‐DAT+NIR group was significantly lower than that in the other two groups (Figure 6H).

### 2.9 Mild Hyperthermia Facilitates Early In Vivo Immune Phenotype Transformation and Neovascularization

**Figure 7:**
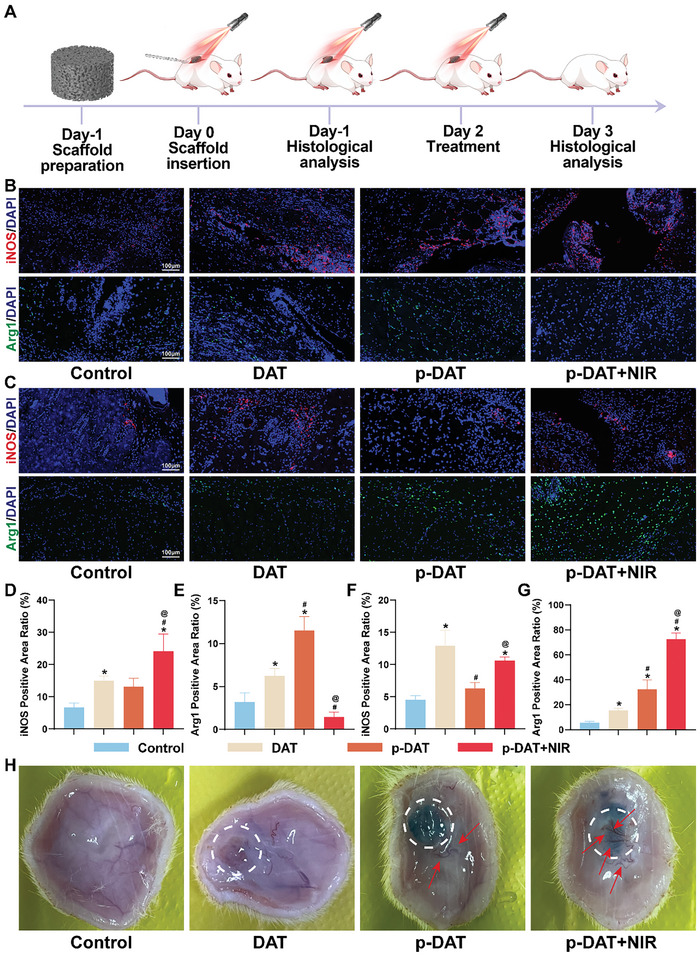
Mild hyperthermia promotes early in vivo immune phenotype transformation and angiogenesis. A) Schematic diagram of the subcutaneous embedding experiment. B) Immunofluorescence staining of macrophage immune phenotypes in the tissue surrounding the scaffold 1 day after subcutaneous embedding. C) Immunofluorescence staining of macrophage immune phenotypes in the tissue surrounding the scaffold 3 days after subcutaneous embedding. D) Immunofluorescence quantitative analysis of iNOS 1 day after subcutaneous embedding (n = 3). E) Immunofluorescence quantitative analysis of Arg1 1 day after subcutaneous embedding (n = 3). F) Immunofluorescence quantitative analysis of iNOS 3 days after subcutaneous embedding (n = 3). G) Immunofluorescence quantitative analysis of Arg1 3 days after subcutaneous embedding (n = 3). H) Macroscopic image of neovascularization around the scaffold (red arrows represent newly formed blood vessels). (^*^ *p* < 0.05 compared to the Control group, ^#^ *p* < 0.05 compared to the DAT group, ^@^ *p* < 0.05 compared to the p‐DAT group).

**Figure** **7**A illustrates a schematic representation of the subcutaneous embedding experiment, in which the scaffold was implanted into the body. Post‐operatively, the first phototherapy session was conducted, followed by continuous illumination for 3 days (15 min per day) (Figure S18, Supporting Information). Skin tissues directly above the scaffold were collected for immunofluorescence staining after 1 and 3 days of mild hyperthermia treatment. Figure 7B,C illustrate the macrophage immune phenotypes in skin tissue following mild hyperthermia treatment for 1 day and 3 days, respectively. In comparison to the CON group, the DAT group demonstrated increased expression of both iNOS and Arg1, indicating that DAT induces a special immune response when used in vivo as a graft. The p‐DAT group exhibited reduced iNOS expression and increased Arg1 expression relative to the DAT group, largely attributed to the anti‐inflammatory effects of PDA loaded onto p‐DAT, as documented in previous studies.^[^ ^25^, ^37^ ^]^ This resulted in a reduced number of M1 macrophages and increased number of M2 macrophages following implantation. In the p‐DAT+NIR group, iNOS expression increased, whereas Arg1 expression was reduced compared to that in the p‐DAT group. This aligned with the findings from in vitro experiments and further confirmed the early effects of mild hyperthermia on macrophage phenotype in vivo. After 3 days of continuous mild hyperthermia treatment, the patterns observed in the initial three groups remained consistent with those observed on the first day. However, overall Arg1 expression levels were higher than those on the first day, indicating a transition from the inflammatory phase to the repair phase. These alterations were significant in the p‐DAT+NIR group. Although iNOS expression remained higher than that in the p‐DAT group, it reduced compared to that on the initial day. Additionally, Arg1 levels were significantly higher in the p‐DAT+NIR group than in the p‐DAT group, indicating an earlier and enhanced repair phenotype that is beneficial for tissue repair. Quantitative fluorescence experiments further substantiated the significance of these observed differences (Figure 7D–G).

Neovascularization plays a crucial role in bone tissue repair by facilitating nutrient supply and waste removal.^[^ ^38^ ^]^ Therefore, in addition to subcutaneous embedding experiments, we assessed the effect of mild hyperthermia on early vascularization. Macroscopic images demonstrated the excellent proangiogenic properties of DAT scaffolds. Compared to the CON group, all scaffold implantation groups exhibited significant neovascularization around the scaffolds. However, in the DAT and p‐DAT groups, vessels did not penetrate the scaffolds. In contrast, after 3 days of continuous mild hyperthermia treatment, the p‐DAT+NIR group displayed a significantly higher number of newly formed vessels around the scaffolds compared to that of the other groups and exhibited extensive vascular ingrowth into the scaffolds (Figure 7H). These findings highlight the potent effects of mild hyperthermia treatment in facilitating in vivo neovascularization.^[^ ^12^, ^39^ ^]^

### 2.10 Mild Hyperthermia Facilitates In Vivo Bone Defect Regeneration

**Figure 8:**
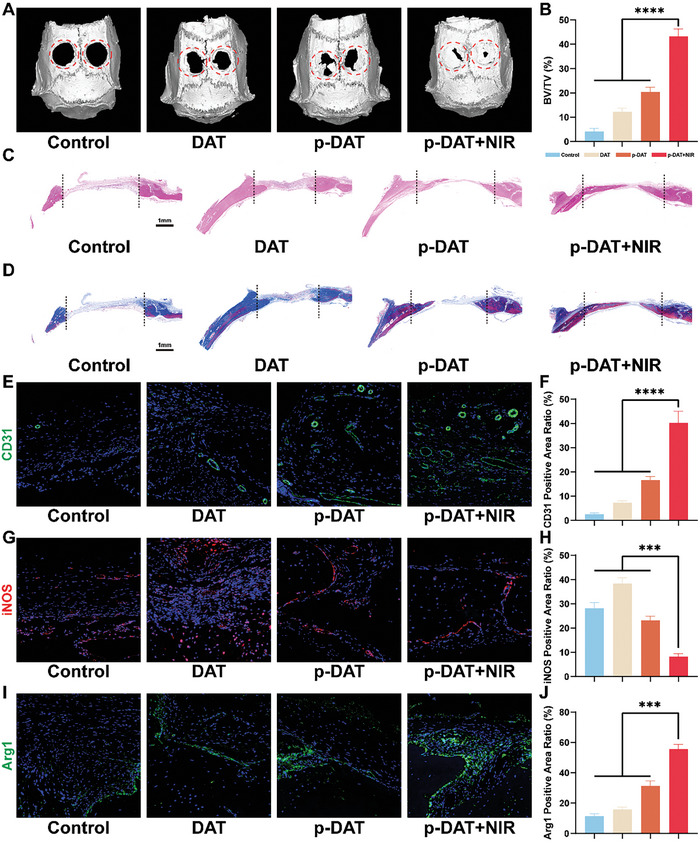
Mild hyperthermia promotes in vivo bone defect restoration. A) Micro‐CT images of new bone formations in the cranial bone defects of the rats in 8 weeks post‐surgery (red dashed circles represent the modeled area of the bone defect). B) Bone volume/tissue volume (BV/TV) ratios at the defect in 8 weeks (n = 3). C) The H&E staining images of new bone formation after implantation for 8 weeks (black dashed lines represent the modeled area of bone defect). D) The Masson staining images of new bone formation after implantation for 8 weeks (black dashed lines represent the modeled area of bone defect). E) Immunofluorescent staining images for CD31. F) Quantitative analysis of CD31 positive area (n = 3). G) Immunofluorescent staining images for iNOS. H) Quantitative analysis of iNOS positive area. I) Immunofluorescent staining images for Arg1 (n = 3). J) Quantitative analysis of Arg1 positive area (n = 3). (^***^ *p* < 0.001, ^****^ *p* < 0.0001).

We created two critical circular defects, each with a 5 mm diameter, symmetrically along the cranial suture of the rats, and subsequently, the material was implanted into these defect sites (Figures S19 and S20, Supporting Information). Following surgery, NIR irradiation treatment was administered to Sprague–Dawley (SD) rats for 3 consecutive days, excluding the p‐DAT group (Figure S21, Supporting Information). Subsequently, they were fed normally for ≈8 weeks before being euthanized for sampling. As illustrated in **Figure** **8**A, the critical cranial defects in the p‐DAT+NIR group were nearly completely repaired with newly formed bone, whereas the cavities in the p‐DAT group were only partially filled. Substantial cavities remained were observed in the CON and DAT groups. Volume quantification of the newly formed bone revealed that the bone volume/total volume ratio in the p‐DAT+NIR group was significantly higher than that in the CON, DAT, and p‐DAT groups (Figure 8B). The osteogenic, angiogenic, and immunoregulatory effects in vivo were assessed through HE, Masson, and immunofluorescence staining of CD31, iNOS, and Arg1. HE staining images revealed significant novel bone regeneration in the p‐DAT+NIR group, accompanied by robust matrix production in the defect area (Figure 8C). Masson staining revealed an abundant and dense collagen matrix in the p‐DAT+NIR group, indicating active osteogenesis, whereas only a scattered collagen matrix is present in other groups (Figure 8D). Immunofluorescent staining of CD31 indicated a significantly higher density of newly formed vessels in the defect areas of the p‐DAT+NIR group compared to those in the other groups (Figure 8E). Quantitative analysis of the CD31‐positive area ratio demonstrated a significant increase in this ratio in the p‐DAT+NIR group than that in the other groups (Figure 8F).

Immunofluorescent staining of iNOS and Arg1 demonstrated that 8 weeks after scaffold implantation, the p‐DAT+NIR group exhibited the lowest iNOS fluorescence intensity (Figure 8G) and highest Arg1 fluorescence intensity (Figure 8I). These findings indicate that after 8 weeks of repair, the immune phenotype of the p‐DAT+NIR group was favorable for tissue repair, aligning with its bone repair status. Quantitative immunofluorescence analysis confirmed that the fluorescence intensities of iNOS and Arg1 were significantly lower and higher, respectively, in the p‐DAT+NIR group than in the other three groups (Figure 8H,J). We harvested and analyzed the hearts, livers, spleens, lungs, kidneys, and brains of rats in each group. The results exhibited no significant differences between groups, indicating that DAT scaffolds and mild hyperthermia treatment were biologically safe and nontoxic to animals (Figure S22, Supporting Information).

The limitations of this study are as follows: 1) We did not extend our investigation to explore the connections between other amino acid metabolic pathways and bone immune regulation; 2) The materials developed in the present study are only suitable for repairing bone defects in non‐load‐bearing regions. Future research should delve deeper into the interplay between amino acid metabolism and immune regulation, with a particular emphasis on critical organelles such as mitochondria and the endoplasmic reticulum. Additionally, there is a need to design bone repair implants that can meet the mechanical demands of load‐bearing applications.

## 3 Conclusion

Overall, this study assessed the beneficial effects of mild hyperthermia treatment on bone repair, highlighting its regulatory effect on immune cells in the early stages following bone defect surgery. These findings indicate that mild hyperthermia dynamically regulates macrophages rather than maintaining a static effect. After 1 day of mild hyperthermia treatment, there was a significant upregulation and downregulation of the *iNOS* and *Arg1* genes, respectively, indicating a pro‐inflammatory phenotype. Following continuous treatment for 3 days, the *iNOS* gene remained highly expressed, whereas *Arg1* expression significantly increased. This indicates that early and sustained photothermal therapy aligns with the inflammatory phase of bone healing and facilitates a rapid transition to the repair phase. RNA‐Seq revealed that immune alterations induced by 1 day of mild hyperthermia treatment align with the early inflammatory phase of bone repair, characterized by osteoclast activation, cell recruitment, and neovascularization, thereby preparing for a rapid transition to the repair phase. The present research highlights the dual temporal effects of early mild photothermal therapy on immune regulation and provides recommendations for the optimal initiation time and duration of photothermal therapy following bone repair surgery. Additionally, we developed a straightforward and efficient photothermal bone tissue engineering material that offers valuable insights into the timing and duration of NIR therapy following bone defect surgery.

## 4 Experimental Section

### Cell culture

RAW264.7 macrophages (RAW), MC3T3‐E1 subclone 14 (MC‐14), and human umbilical vein endothelial cells (HUVEC) were obtained from the Cell Bank of the Chinese Academy of Science (Shanghai, China). RAW and MC‐14 cells were cultured in α‐MEM (Gibco, USA) supplemented with 1% penicillin‐streptomycin (P/S; Gibco, USA) and 10% fetal bovine serum (FBS; Gibco, USA). HUVECs were cultured in an Endothelial Cell Medium (ECM; ScienCell, USA). All cells were maintained at 37 °C in a humidified atmosphere with 5% CO_2_. For the mild hyperthermia treatment group, cells were placed in an incubator at 41.5 ± 0.5 °C for 15 min each day. For convenience, the control group in the in vitro experiments was referred to as the CON group, while the mild hyperthermia group was referred to as the HEAT group.

### Biocompatibility Testing

RAW and MC‐14 cells were seeded in 96‐well plates at a density of 5000 cells per well and divided into CON and HEAT groups. The HEAT group was placed in an incubator at 41.5 ± 0.5 °C for 15 min each day, with the culture medium replaced daily. After 1 and 3 days of culture, 100 µL of culture medium containing 10% CCK‐8 reagent (Dojindo, Japan) was added to each well and incubated for 2 h, after which the absorbance at 450 nm was measured using a microplate reader. To evaluate cytotoxicity, live/dead staining was conducted. Live cells, stained with calcein‐AM (Beyotime Biotechnology, China), showed green fluorescence, while dead cells, stained with PI (Beyotime Biotechnology, China), showed red fluorescence.

Cell apoptosis was detected using flow cytometry. Briefly, cells were resuspended in 300 µL of Binding Buffer (1×, BD Pharmingen, USA), followed by the addition of 5 µL of FITC‐labeled Annexin V (BD Pharmingen, USA). The mixture was incubated in the dark at room temperature for 15 min, after which 5 µL of Propidium Iodide (BD Pharmingen, USA) was added and incubated for 5 min. Finally, 200 µL of 1× Binding Buffer was added and mixed well before detection and analysis using a flow cytometer.

### Cell Migration Assay

MC‐14 cells were seeded into 6‐well plates at a density of 20 000 cells per well. Once the cells reached full confluence, a straight line was scratched along the diameter of each well using a 200 µL pipette tip. The wells were then rinsed with PBS to remove any detached cells and photographed under a light microscope to record the initial wound. After a 24 h incubation period, the wells were photographed again under the light microscope to document the wound healing progress. In the Transwell experiment, MC‐14 cells were seeded at different densities (10 000, 20 000, and 50 000 cells per insert) in the upper chambers of 24‐well Transwell plates with 8 µm pore size. After incubation for 1 day, the cells were fixed with 4% paraformaldehyde (Servicebio, China) and stained with crystal violet (Beyotime Biotechnology, China) staining solution for 30 min. Subsequently, the upper chambers were gently wiped with a cotton swab, rinsed with PBS, and photographed under an optical microscope for documentation.

### ROS Detection

DCFH‐DA (Beyotime Biotechnology, China) fluorescent staining and flow cytometry were used to detect the level of ROS under different condition, according to the manufacturer's instructions.

### Macrophage Polarization Experiment

Macrophage polarization‐related gene expression was measured using RT‐qPCR.

(EZBioscience) following the specific operational steps described in a previously published study.^[^ ^25b^ ^]^ The primers used are listed in Table S1 (Supporting Information). The data were evaluated using a QuantStudio 7 Flex system (Life Technologies). Macrophage polarization‐related mRNA levels (mouse‐derived) of *Tnf ‐α*, *iNOS*, *Il1b*, *Il6*, *Cd86*, *Arg1*, *Il1rn*, *Il10*, *Tgfb3*, and *Cd206* were calculated using the 2^−ΔΔCt^ method.

Cell phenotypes were analyzed using flow cytometry to detect surface markers of M1 (CD86, BioLegend, APC anti‐mouse CD86 Antibody) and M2 (CD206, PE anti‐mouse CD206 Antibody) macrophages. Isotype control antibodies for CD206 (PE Rat IgG2a, κ Isotype Ctrl Antibody) and CD86 (APC Rat IgG2a, κ Isotype Ctrl Antibody) were used. Flow cytometry assays and data analysis were performed using a Guava easyCyte Flow Cytometer (Millipore, USA) and CytExpert software, respectively.

The polarization status of macrophages through immunofluorescence staining experiments were further evaluated. The wells were added with 4% paraformaldehyde, fixed at room temperature for 30 min, and washed thrice with PBS for 5 min. Next, 0.5% Triton X‐100 and 5% BSA were used for permeabilization and non‐specific binding site blocks. Primary antibodies against inducible nitric oxide synthase (iNOS, Abcam, 1:500) and arginase‐1 (Arg1, Abcam, 1:200) diluted with immunostaining dilution buffer (Beyotime Biotechnology, China), and incubated overnight at 4 °C. The cells were then incubated with the goat anti‐rabbit Alexa Fluor594 (Abcam, 1:200) or Alexa Fluor488 (Abcam, 1:200) secondary antibodies at room temperature for 1 h to target iNOS or Arg1, respectively. After incubation, the wells were rinsed with PBS, and DAPI (Beyotime Biotechnology, China) was added to stain cell nuclei. The cells were then washed three times with PBS. Staining was observed using a fluorescence microscope (DMI8, Leica, Germany).

Total protein was extracted at 4 °C using RIPA lysis buffer containing protease and phosphatase inhibitors (Solarbio, China). Protein concentration was determined with a BCA Protein Assay Kit (Beyotime, China). Equal amounts of protein were separated by 10% (w/v) SDS‐PAGE and subsequently transferred to a polyvinylidene difluoride membrane (Millipore, USA). After blocking with 5% (w/v) skim milk, the membrane was incubated overnight at 4 °C with primary antibodies, followed by incubation with horseradish peroxidase (HRP)‐conjugated secondary antibodies (1:10 000; 115‐035‐003, 111‐035‐003, Jackson ImmunoResearch, USA) at 25 °C for 1 h. Enhanced chemiluminescence reagent (Solarbio, China) was used to visualize the immunoreactive bands, and the gray values of the protein bands were semi‐quantified using ImageJ software (version 1.48, USA). The primary antibodies used in this study included iNOS (ab178945, Abcam, UK) and anti‐Arg1 (ab96183, Abcam, UK).

### Macrophage Bacterial Phagocytosis Assay

The Staphylococcus aureus suspension was co‐cultured with macrophages for 1 h. Lysostaphin was then used to lyse extracellular bacteria outside the macrophages. After multiple centrifugation steps to remove dead bacteria, macrophages were lysed with 0.1% Triton X‐100 for 15 min to release intracellularly engulfed bacteria. The resulting supernatant was used for plating assays to assess the macrophage's ability to phagocytose bacteria.

### In Vitro Osteogenesis

The osteogenic induction culture medium contains 10% FBS, 100 U mL^−1^ P/S, 50 mg L^−1^ ascorbic acid, 10 mM 𝛽‐glycerophosphate, and 10^−8^ m dexamethasone. All reagents were purchased from Gibco (Carlsbad, CA, USA). ALP (Beyotime Biotechnology, China) and ARS (Cyagen, USA) were performed after 7 and 14 days, respectively. For the quantitative analysis of ARS, 10% cetylpyridinium chloride was prepared with PBS, and 500 µL of the solution was added to each well to replace PBS. Results were recorded using a 570 nm microplate reader (Bio‐Rad) for statistical analysis.

### Transcriptome Analysis

RNA was extracted from RAW264.7 macrophages treated with or without mild hyperthermia, using the EZ‐press RNA Purification Kit (EZbioscience, USA) according to the manufacturer's instructions. The library construction and sequencing were carried out at Shenzhen BGI Genomics Co. The Limma package was used for differential gene expression analysis of the sequencing data (threshold: |logFC| > [mean(|logFC|) + 2sd(|logFC|)], *p* < 0.05) to identify genes affected through heat stimulation. The clusterProfiler package was used for the enrichment analysis of biological processes and GSEA of differentially expressed genes; the Metascape platform (https://metascape.org/) was utilized for the construction and visualization of differentially expressed gene enrichment networks; the String database (https://cn.string‐db.org/) was employed to construct the protein–protein interaction network of differentially expressed genes.

### Preparation of DAT

Pig subcutaneous fat was cut into small pieces and subjected to a decellularization process following previously reported protocols. Initially, the tissue underwent three freeze‐thaw cycles, alternating between −80 and 37 °C. The tissue was then homogenized, rinsed with PBS three times, and digested overnight with 0.25% trypsin/0.02% EDTA. Next, 99.9% isopropanol was used to extract fat. After three additional PBS washes, the tissue was incubated for 4 h in the enzymatic solution, followed by three more PBS washes. It was then treated with 5000 U deoxyribonuclease, 10 mg ribonuclease, and 2000 U lipase for 6 h at 37 °C. After three more PBS washes, an 8 h isopropanol extraction was performed. Finally, the DAT was homogenized in 75% ethanol, vacuum‐filtered to form a membrane, air‐dried, and stored under vacuum. HE, Masson, Oil Red, and DAPI staining were utilized to evaluate the composition of adipose tissue and DAT.

### Preparation and Characterization of p‐DAT

An appropriate mass of DAT was precisely measured and homogenized to prepare a 5 mg mL^−1^ solution using ultrapure water. Subsequently, different weights of DA powder were carefully diluted in 10 mL of the DAT solution, resulting in DAT/DA mixtures with varying DA concentrations of 0, 0.5, 1, 2, and 4 mg mL^−1^. The pH of the DAT/DA solution was promptly adjusted to 8.5 using Tris buffer and incubated at room temperature for 1 h. DAT and p‐DAT scaffolds were prepared, freeze‐dried, and stored for future use. The photothermal characteristics of p‐DAT scaffolds were evaluated using 808 nm NIR irradiation. FTIR (Thermo Fisher, IS5, USA) and SEM (Merlin Carl Zeiss Jena, Germany) were employed to analyze the chemical composition of DAT and p‐DAT scaffolds.

### Stability of p‐DAT

The p‐DAT scaffolds with or without NIR, were immersed in a PBS solution and subsequently incubated at room temperature for 24 h. Then, UV–vis absorption spectroscopy was employed to measure the concentration of PDA in the supernatant.

### Animal Experiments

Sprague‐Dawley rats (8–12 weeks, male) were purchased from Tongji University Animal Department. All experiments were approved by the Institutional Animal Care and Use Committee (IACUC) of the Tongji University School of Medicine affiliated with Shanghai Tenth People's Hospital (SHDSYY‐2024‐2530). All experimental procedures were performed following the administration of isoflurane anesthesia.

### In vivo bacteriostatic assay

The DAT and p‐DAT scaffolds were immersed in a 1 × 10^5^ concentration of Staphylococcus aureus bacterial solution for 1 h. Subsequently, subcutaneous pockets were created on the backs of SD rats, and the scaffolds mixed with Staphylococcus aureus were implanted into the pockets. The rats were divided into three groups: DAT group, p‐DAT group, and p‐DAT+NIR group, with the DAT and p‐DAT+NIR group receiving photothermal therapy for 15 min daily. After 3 days, samples were collected. First, the scaffolds from each group were removed and vertically slid from top to bottom on a blood agar plate using sterile tweezers, then incubated in a 37 °C incubator for 1 day to observe the bacterial growth inside the scaffold. Second, the scaffolds from each group were placed in the center of a blood agar plate and incubated in a 37 °C incubator for 1 day to observe the inhibitory zone radius around the scaffold, indirectly assessing the bacterial content within the scaffold. Finally, the scaffolds were soaked in PBS solution, and the bacteria inside the scaffold were separated into the PBS through ultrasonication. The suspension was then used for plate counting experiments, and the blood agar plates were incubated in a 37 °C incubator for 1 day. Additionally, skin tissues directly above the scaffolds were extracted and fixed in 4% paraformaldehyde, and covered with gauze to prevent the tissue from curling. After fixation, the samples were dehydrated using graded ethanol solutions (70%, 80%, 90%, and 95% ethanol and absolute ethanol). The samples were then immersed in molten paraffin, removed, and placed in embedding molds. After cooling with a condenser, the wax blocks were labeled and trimmed. Paraffin sections were cut at a thickness of 5 µm, stained with Giemsa staining solution, and observed and recorded under an optical microscope.

### Subcutaneous Implantation Experiment

To evaluate the effect of mild hyperthermia on early phenotypic transformation of macrophages in vivo, DAT and p‐DAT scaffolds were implanted into subcutaneous pockets on the backs of rats. The groups were divided into a control group, a DAT group, a p‐DAT group, and a p‐DAT+NIR group. The control group only had the subcutaneous pocket model without any implants. Except for the p‐DAT group, all received daily NIR light irradiation treatment for 15 min. Samples were collected at 1 day and 3 days for in vivo immunofluorescence experiments. Briefly, the skin tissue directly above the scaffold was excised, fixed in 4% paraformaldehyde, and covered with gauze to prevent curling during fixation. After fixation, the samples were dehydrated using 70%, 80%, 90%, and 95% ethanol and absolute ethanol. The samples were then immersed in molten paraffin, removed, and placed in embedding molds. Once cooled, the wax blocks were labeled, trimmed, and sectioned at a thickness of 5 µm. Immunofluorescence staining was performed on the paraffin sections using iNOS and Arg1 antibodies.

### Critical‐Sized Bone Defects Model

After anesthesia, a full‐thickness incision was made along the sagittal suture to create two critical‐sized bone defects (5 mm in diameter) on both parietal bones using a dental drill. A 5 mm diameter scaffold was implanted into the cranial defect site, with groups divided into control, DAT, p‐DAT, and p‐DAT+NIR (Figure S11, Supporting Information). The wound was sutured layer by layer, and postoperative antibiotics were administered to prevent infection, allowing the rats unrestricted activity. Besides the p‐DAT group, all groups received 15 min of photothermal therapy daily for 3 days. After 2 months, the rats were euthanized with excess anesthesia for collection of skull samples.

The harvested skull specimens underwent high‐resolution Micro‐CT scanning using Skyscan 1076 equipment. Scan data were reconstructed using Mimics software and further analyzed with Skyscan CTAn software. After fixing the skulls, they were decalcified in the solution at 37 °C with 180 rpm rotation, changing the solution every 2 days. After 1 month, specimens were embedded in paraffin and sectioned. Sections were subjected to HE and Masson staining to assess newly formed bone tissue and collagen fibers. Observations and photographs were taken under a bright‐field microscope. Immunofluorescence staining for CD31 and DAPI marked cell nuclei to evaluate newly formed blood vessels in paraffin‐embedded sections, observed and photographed under a fluorescence microscope. Immunofluorescence staining for iNOS and Arg1 characterized macrophage immunophenotypes in newly formed bone tissue.

To assess scaffold biocompatibility, samples from the heart, liver, spleen, lungs, kidneys, and brain were collected from different groups. These samples were stained with HE and observed under a bright‐field microscope for further analysis and photography.

### Statistical Analysis

Statistical analyses were conducted using GraphPad Prism 9 software (GraphPad Software, Inc., La Jolla, CA, USA). Data were presented as mean ± standard deviation. Statistical analysis was performed using Student's *t*‐test for binary group comparisons, or one‐way analysis of variance followed by Tukey's post‐hoc‐test for multiple group comparisons (n = 3). Statistical significance was defined as *p* < 0.05.

## Conflict of Interest

The authors declare no conflict of interest.

## Supporting information



Supporting Information

## Data Availability

The data that support the findings of this study are available from the corresponding author upon reasonable request.
