# Supplementary material for: Mild Hyperthermia Accelerates Bone Repair by Dynamically Regulating iNOS/Arg1 Balance in the Early Stage
Source: Adv Sci (Weinh). 2024 Dec 31;12(8):2409882. doi: 10.1002/advs.202409882 (PMC11848644; doi:10.1002/advs.202409882)
Supplement: Supplementary file 1 — Supporting Information [file ADVS-12-2409882-s001.docx]

**Supporting Information**

**Mild hyperthermia accelerates bone repair by dynamically regulating iNOS/Arg1 balance in the early stage**

Jinhui Zhao^1,^**^#^**, Yiping Luo^1,^**^#^**, Lei Zhang^1,^**^#^**, Yunfeng Chen^1^, Yixing Chen^1^, Xinhui Wu^1^, Aihemaitijiang Aierken^1^, Dilixiati Duolikun^1^, Tianlong Wang^1,^*****, Zifei Zhou^1,^*****, Zhiqing Liu^1,^*****, Longpo Zheng^1,2,3,^*****

^1^Department of Orthopedics, Shanghai Tenth People’s Hospital, School of Medicine, Tongji University, Shanghai 200072, China.

^2^Shanghai Trauma Emergency Center, Shanghai 200072, China.

^3^Orthopedic Intelligent Minimally Invasive Diagnosis & Treatment Center, Shanghai Tenth People's Hospital, Tongji University School of Medicine, Shanghai 200072, China

**Table S1** Primers used for q-RT PCR of genes (mouse derived)

| **Genes** | **Upper primer sequence (5’ to 3’)** | **Lower primer sequence (5’ to 3’)** |
| --- | --- | --- |
| ***Tnf-α*** | TAGCCAGGAGGGAGAACAGA | CCAGTGAGTGAAAGGGACAGA |
| ***iNOS*** | TTGACGCTCGGAACTGTA | GTTGGTGGCATAAAGTATGTG |
| ***Il1b*** | TACATCAGCACCTCACAAGC | AGAAACAGTCCAGCCCATACT |
| ***Il6*** | ACCAAGACCATCCAATTCATC | CTGACCACAGTGAGGAATGTC |
| ***Cd86*** | TCTCCAACAGCCTCTCTCTTT | ATCTTCATTGACTCCGTTTCC |
| ***Arg1*** | TGCTCACACTGACATCAACAC | GAGAATCCTGGTACATCTGGG |
| ***Il1rn*** | TGAATCCTGTGACCCTGTG | AAACTGAACCCCTGAGAAGAG |
| ***Il10*** | GGAAAACCTCGTTTGTACCT | GGGCTTCTTTCTAAATAGTTCAC |
| ***Tgfb3*** | TGGTGGTGAAGTCGTGTAAGTG | TGAGGTCTGTCGCTTTGGTT |
| ***Cd206*** | AGGGAAGAGAAGAAGATCCAG | TGGGAGAAGATGAAGTCAAAC |
| ***GAPDH*** | AAATGGTGAAGGTCGGTGTG | AGGTCAATGAAGGGGTCGTT |

The primers were synthesized and provided by BioTNT.


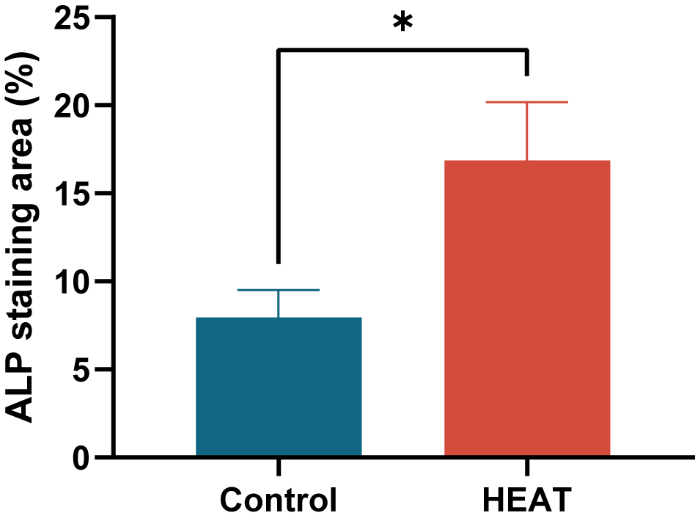


**Figure S1.** Quantitative analysis of ALP staining (n=3, *****P < 0.05).


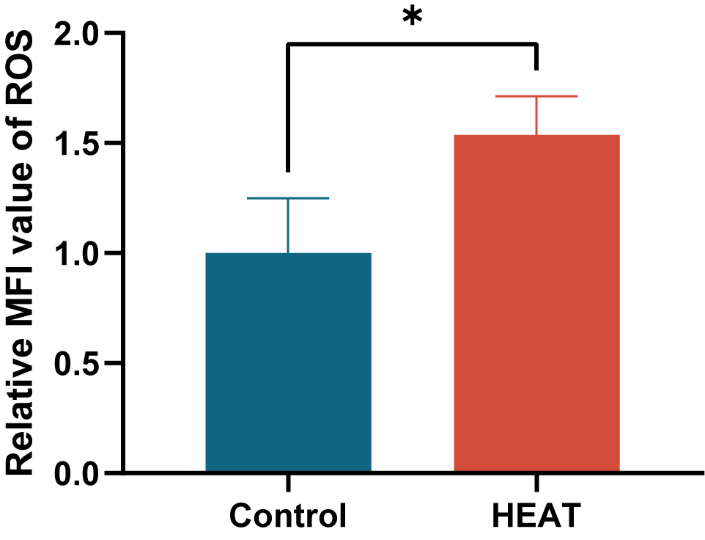


**Figure S2.** Quantitative analysis of ROS staining (n=3, *****P < 0.05).


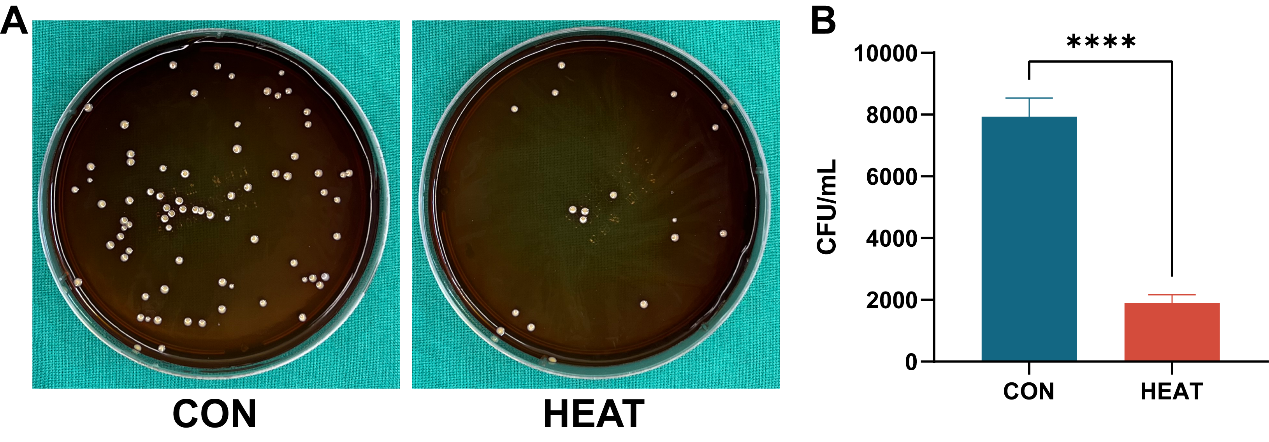


**Figure S3. Evaluation of the antibacterial properties of nitric oxide.** A) Typical photos of *S. aureus* biofilms. B) Counting results of *S. aureus* (n=3, ********P < 0.0001).


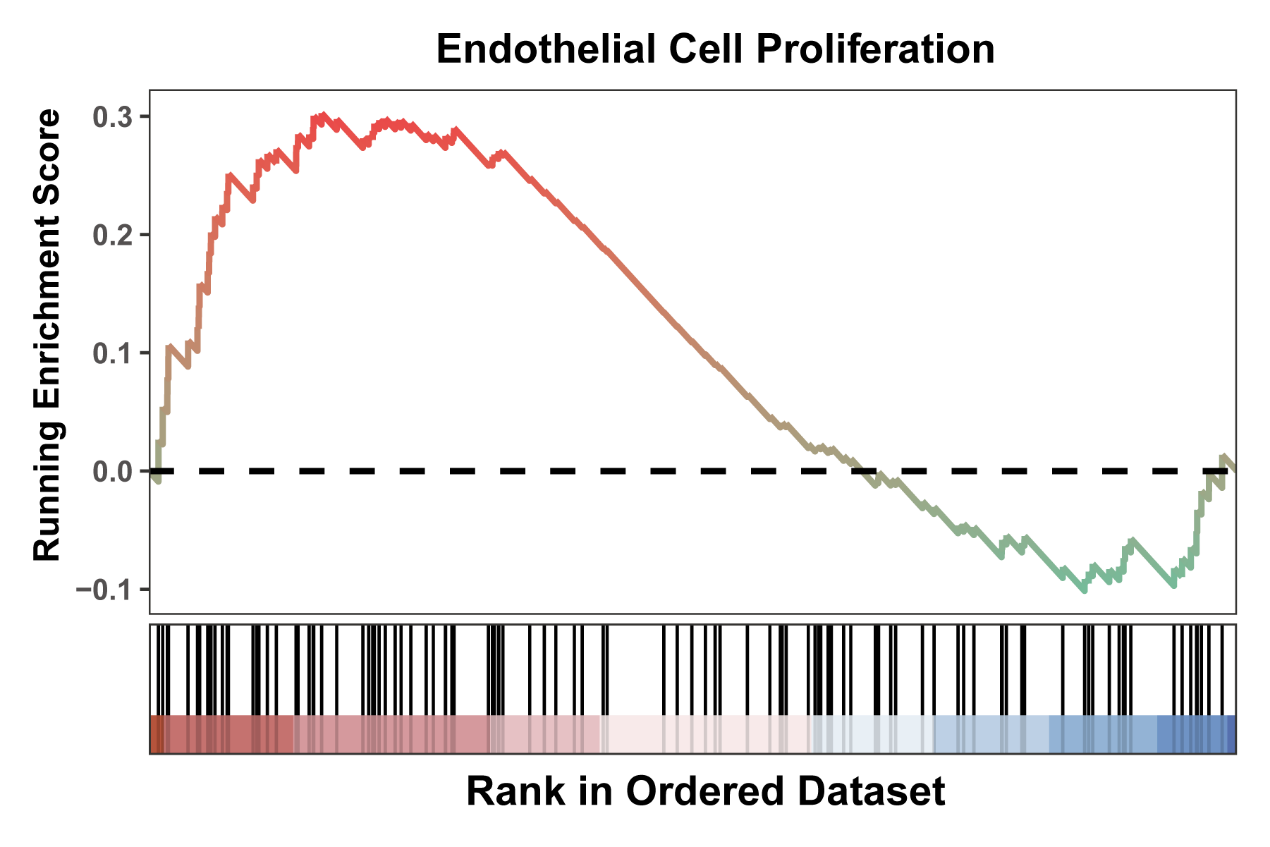


**Figure S4. GSEA analysis of endothelial cell proliferation.**


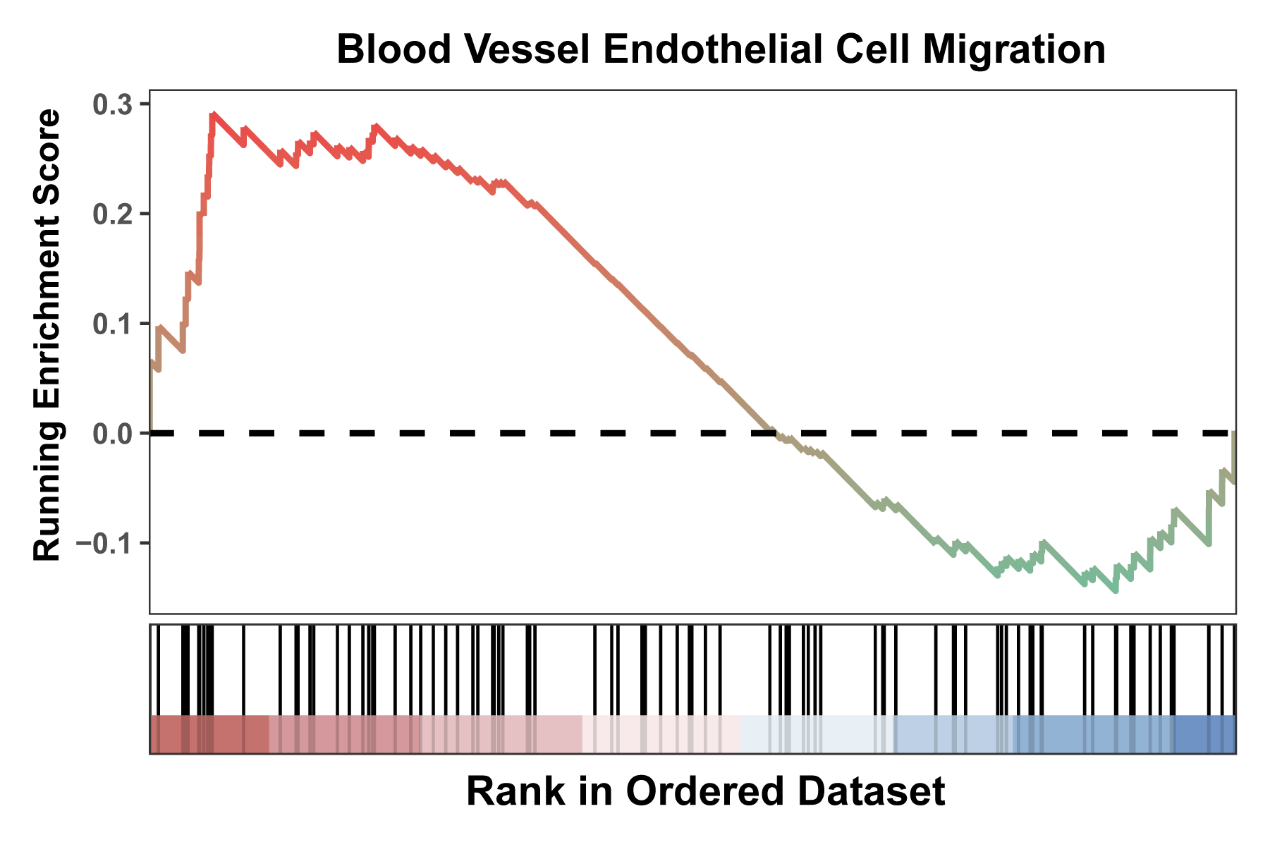


**Figure S5. GSEA analysis of blood vessel endothelial cell migration.**


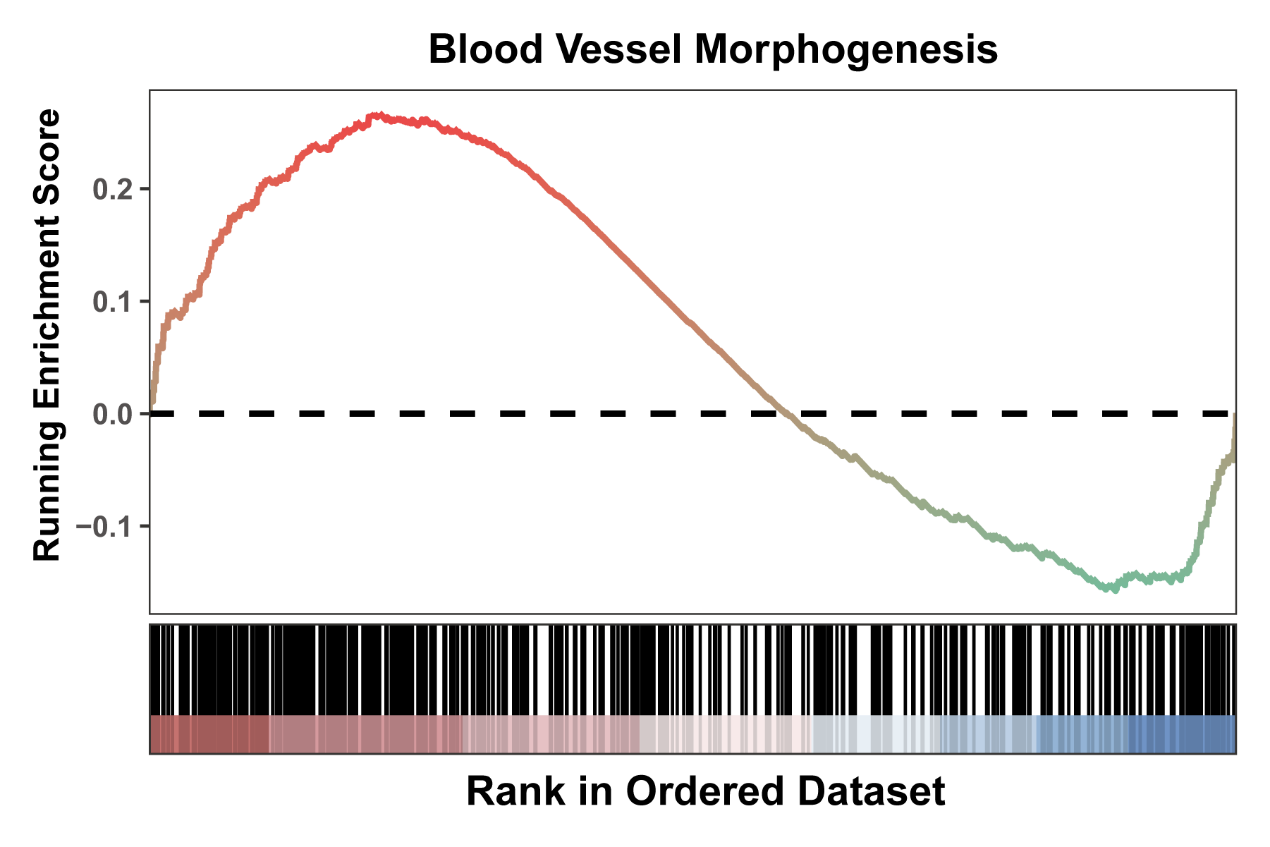


**Figure S6. GSEA analysis of** **blood vessel morphogenesis.**


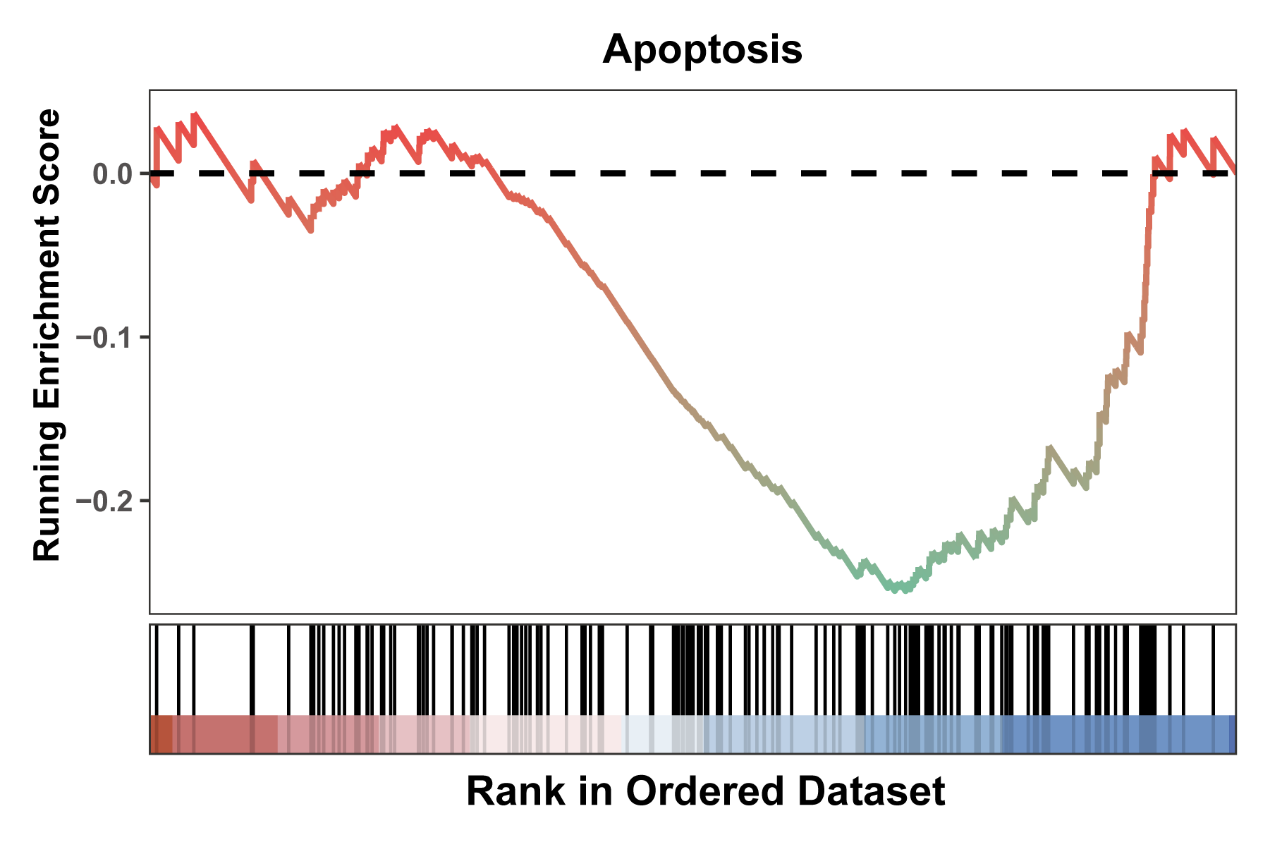


**Figure S7. GSEA analysis of apoptosis.**


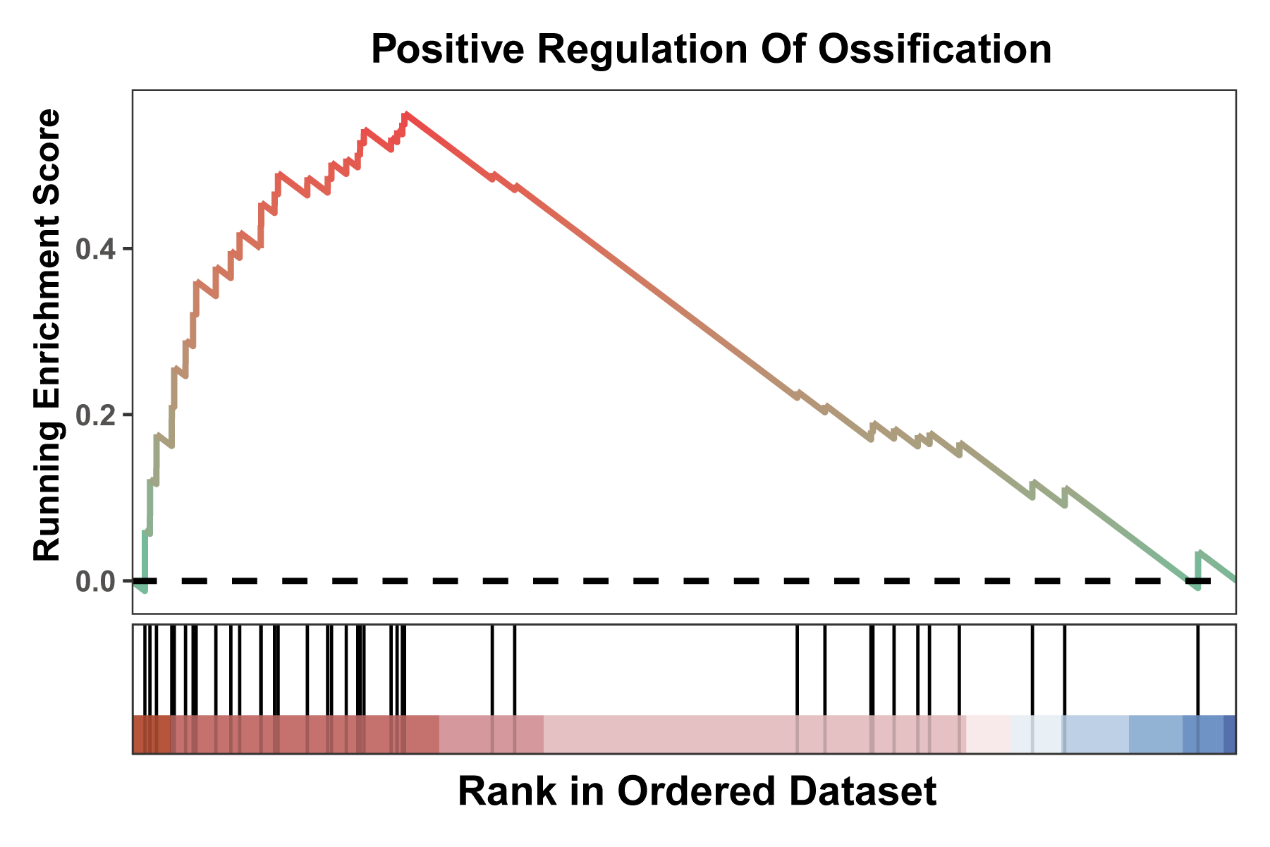


**Figure S8. GSEA analysis of ossification.**


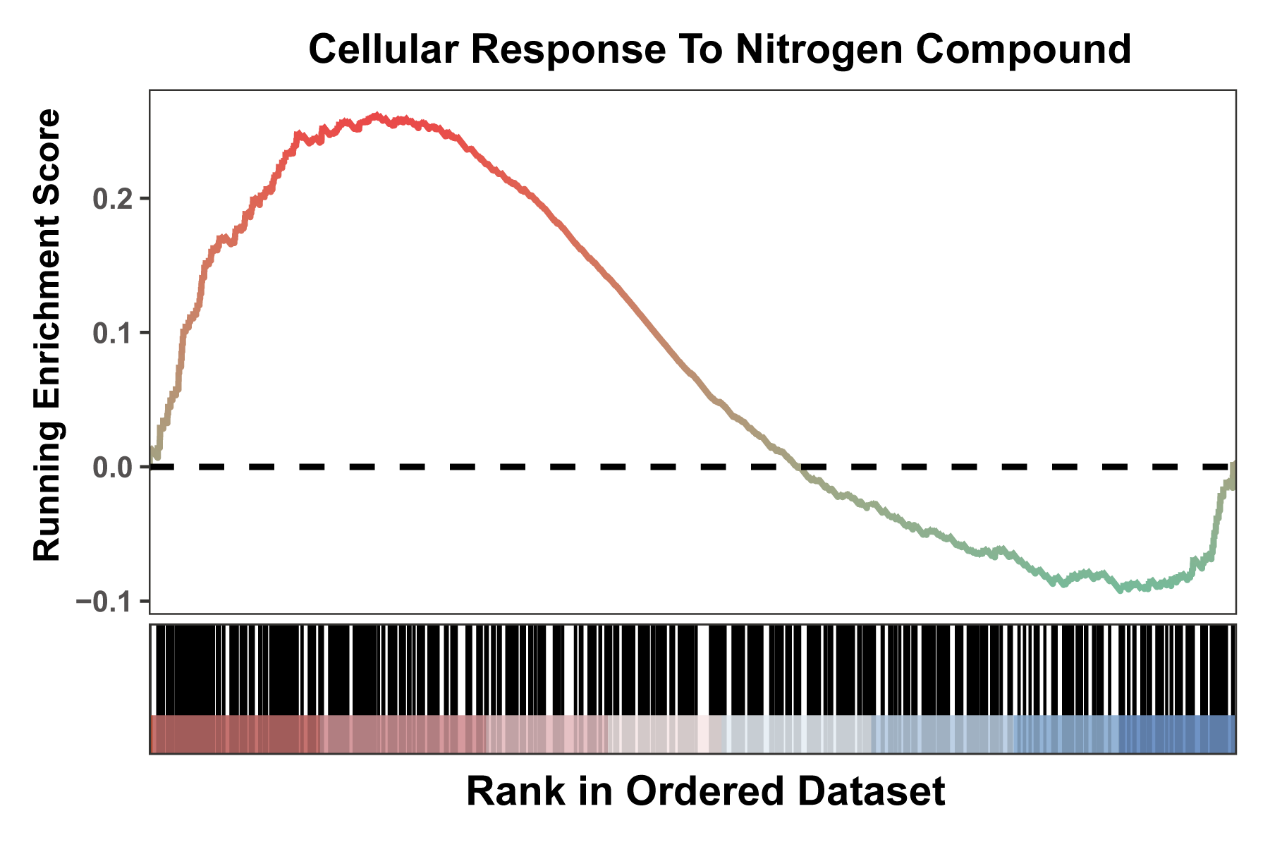


**Figure S9. GSEA analysis of cellular response to nitrogen compound.**


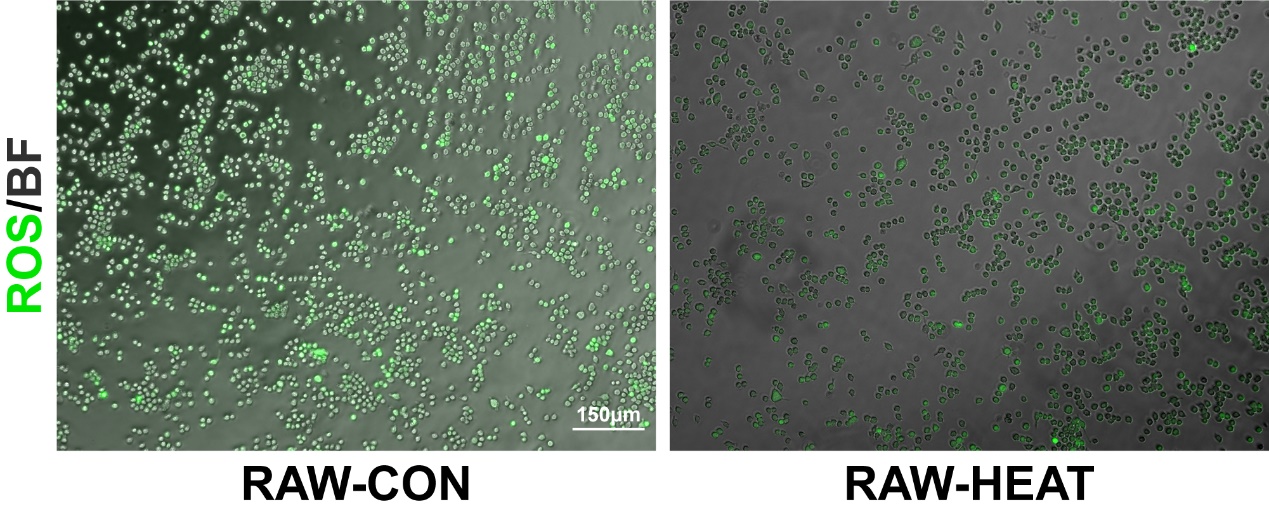


**Figure S10.** ROS fluorescent staining following 3 days of mild hyperthermia treatment.


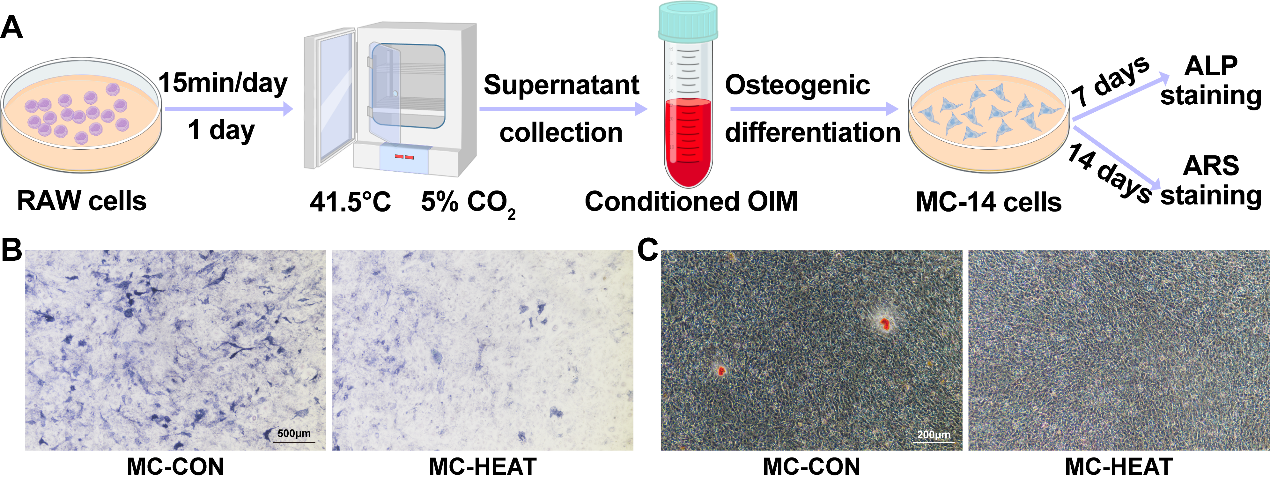


**Figure S11.** **Co-culture of macrophages with osteoblasts after 1 day of mild hyperthermia treatment.** A) Schematic diagram of osteogenic induction conditioned medium configured with macrophage supernatant to co-culture MC-14 cells. B) ALP staining cocultured with immunoregulatory RAW cells. C) ARS staining cocultured with immunoregulatory RAW cells.


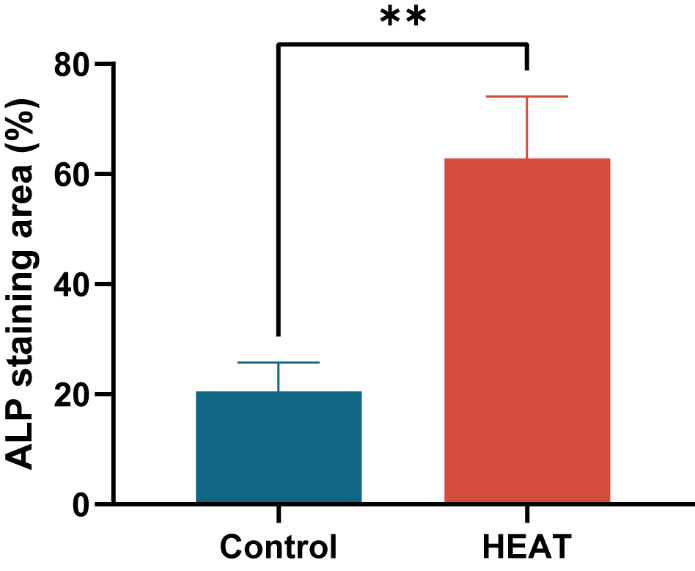


**Figure S12.** Quantitative analysis of ALP staining (n=3, ******P < 0.01).


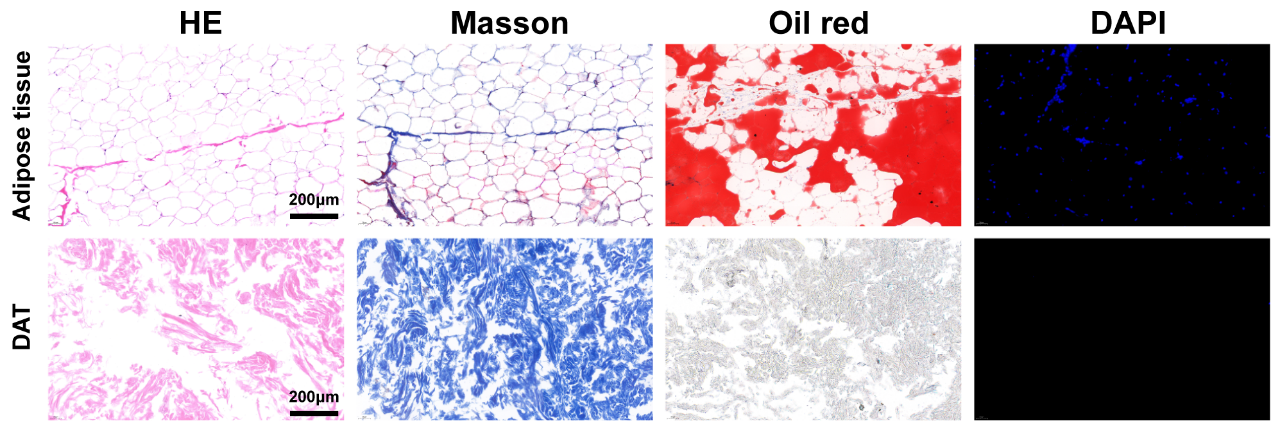


**Figure S13.** Histological examination of adipose tissue and DAT (Hematoxylin and Eosin staining, Masson's Trichrome staining, Oil Red O staining, and DAPI staining).


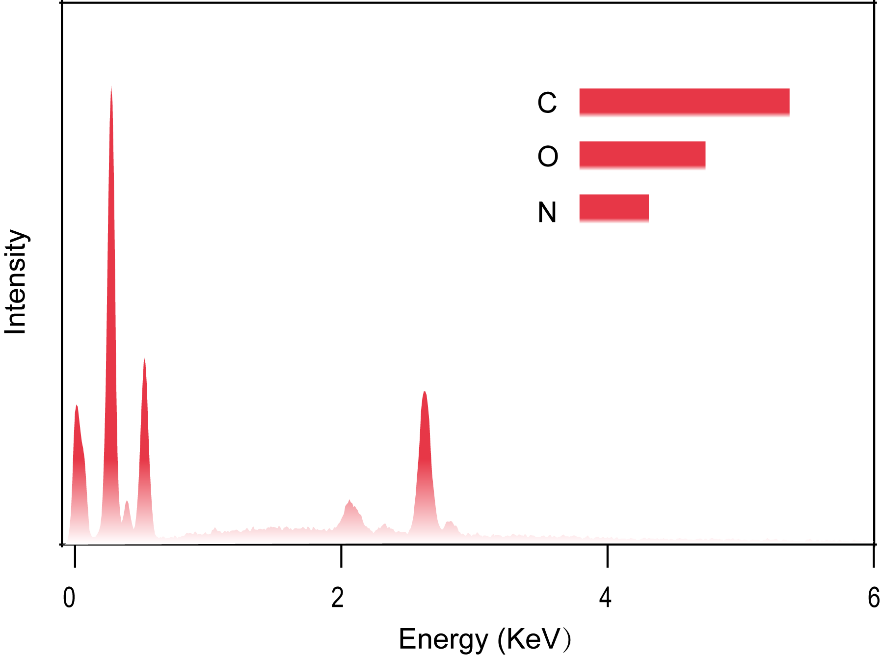


**Figure S14.** EDS of DAT.


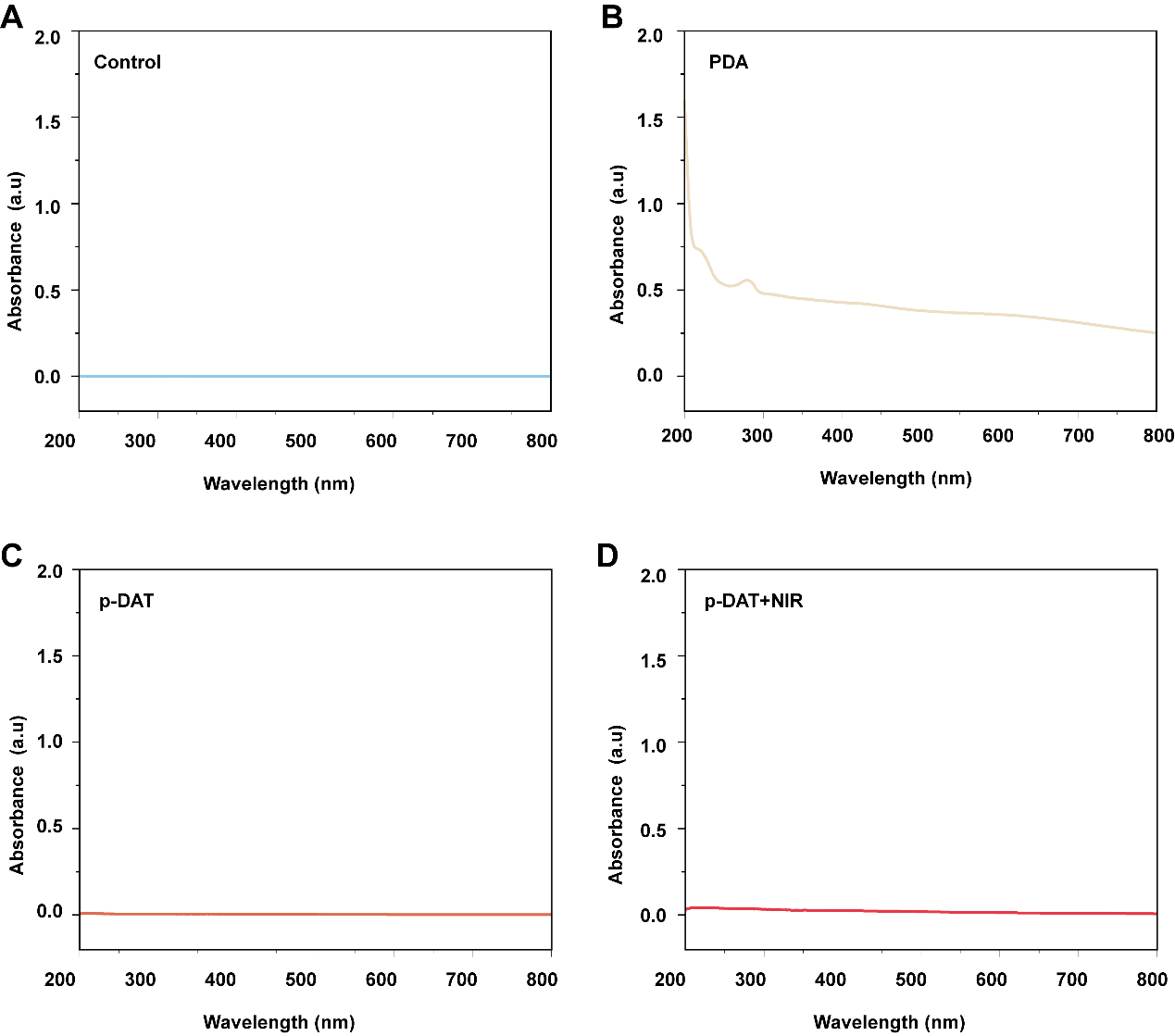


**Figure S15.** UV–Vis of A) PBS, B) PDA, C) p-DAT, and D) p-DAT under 808 nm NIR irradiation.


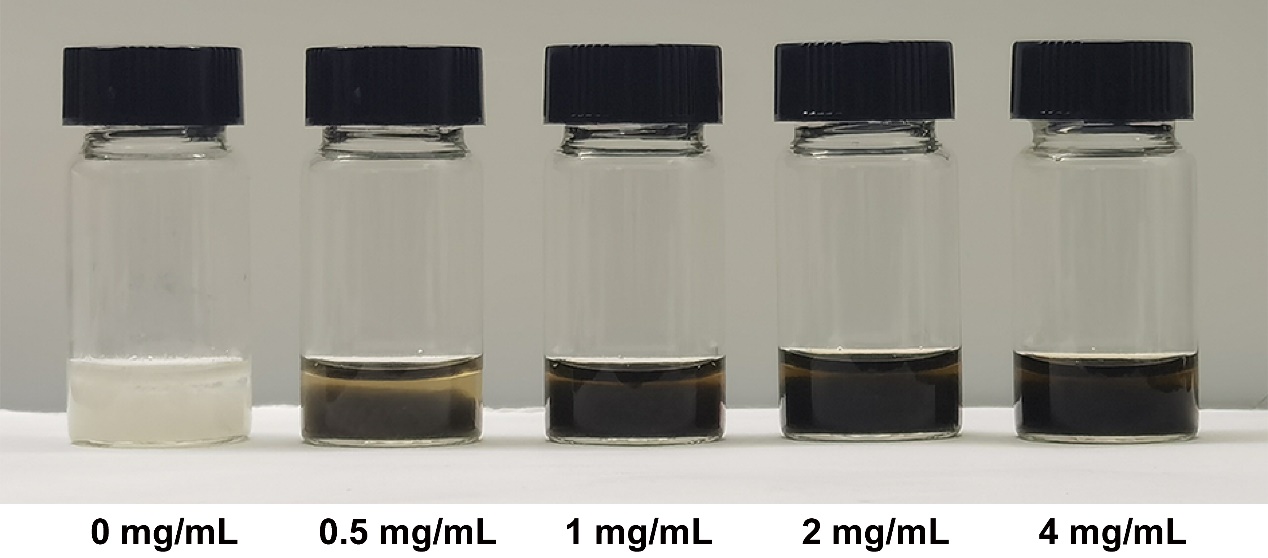


**Figure S16.** Gross observation of DAT and p-DAT with various DA concentrations by digital camera.


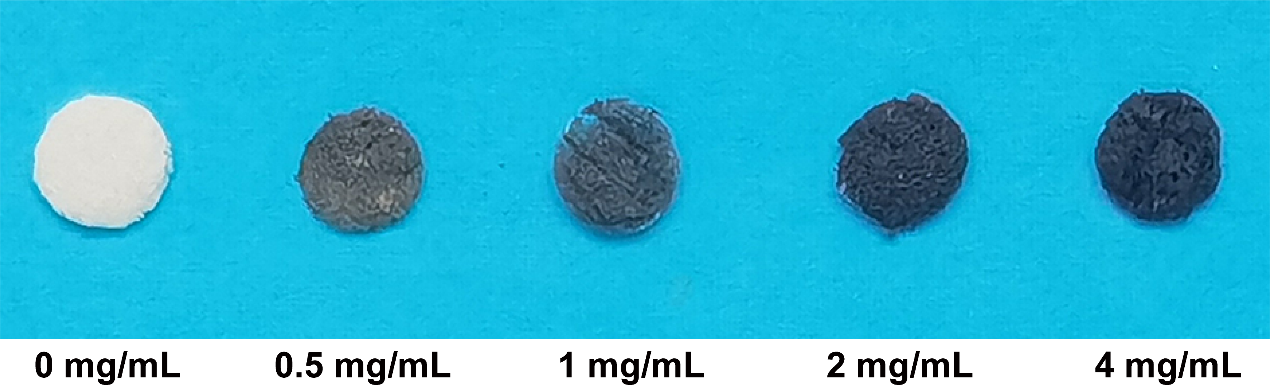


**Figure S17.** Gross observation of DAT and p-DAT with various DA concentrations after freeze-drying.


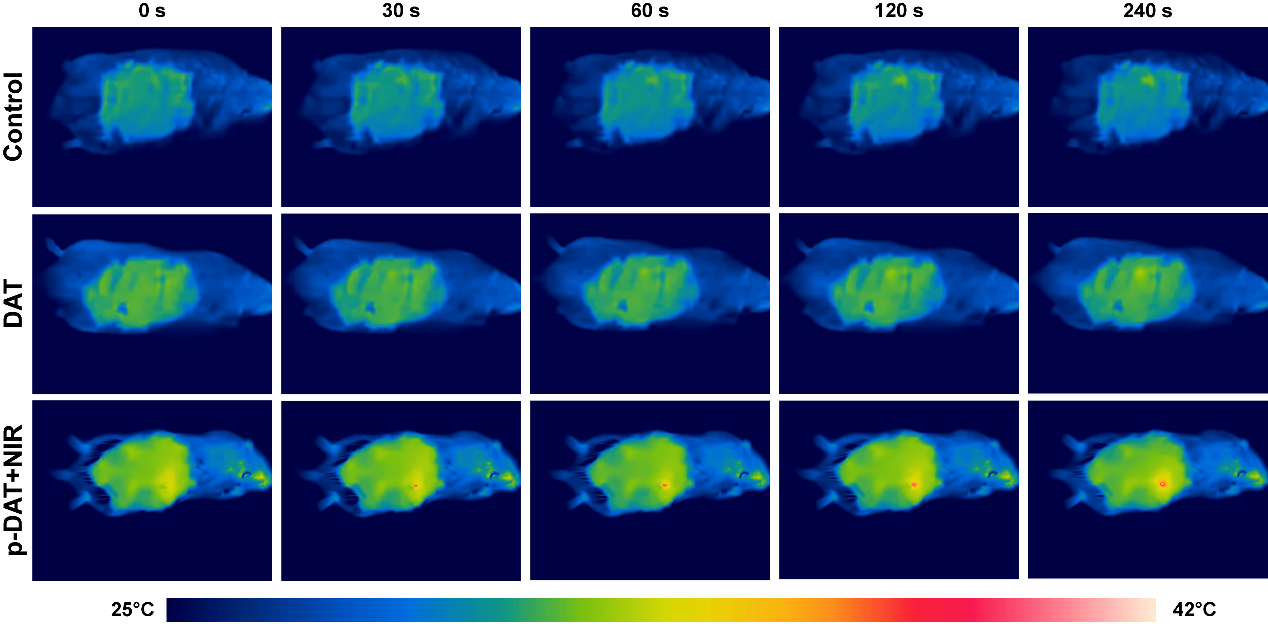


**Figure S18.** Thermal images of pDAT scaffolds implanted subcutaneously under 808 nm NIR irradiation a power density of 0.7 W/cm^2^.


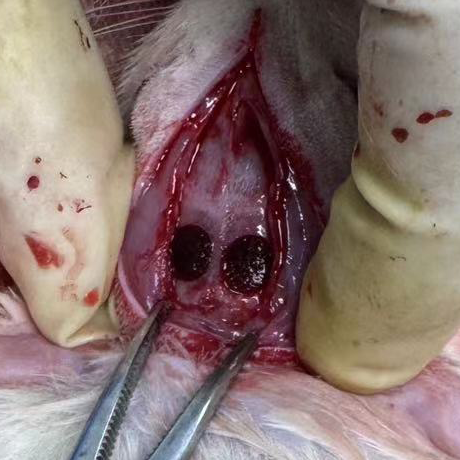


**Figure S19.** Cranial defect modeling in rats.


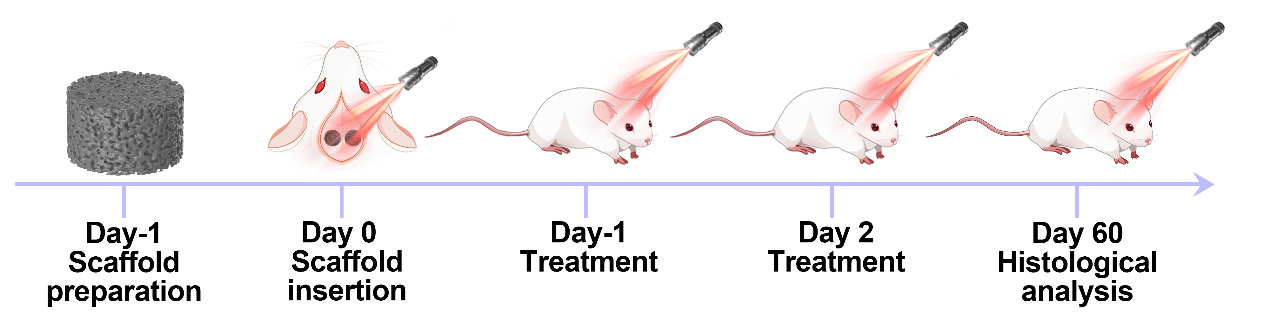


**Figure S20.** Schematic diagram of the bone defect model.


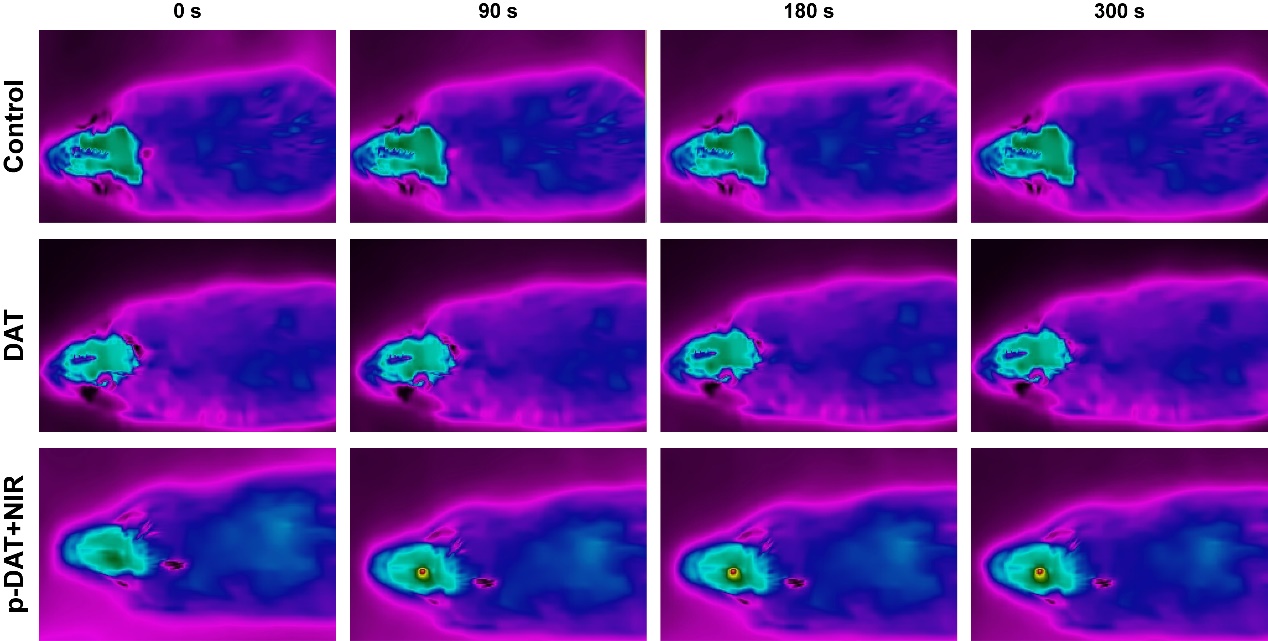


**Figure S21.** Thermal images of p-DAT scaffolds implanted in the 5 mm of skull defect site under 808 nm NIR irradiation with a power density of 0.7 W/cm^2^.


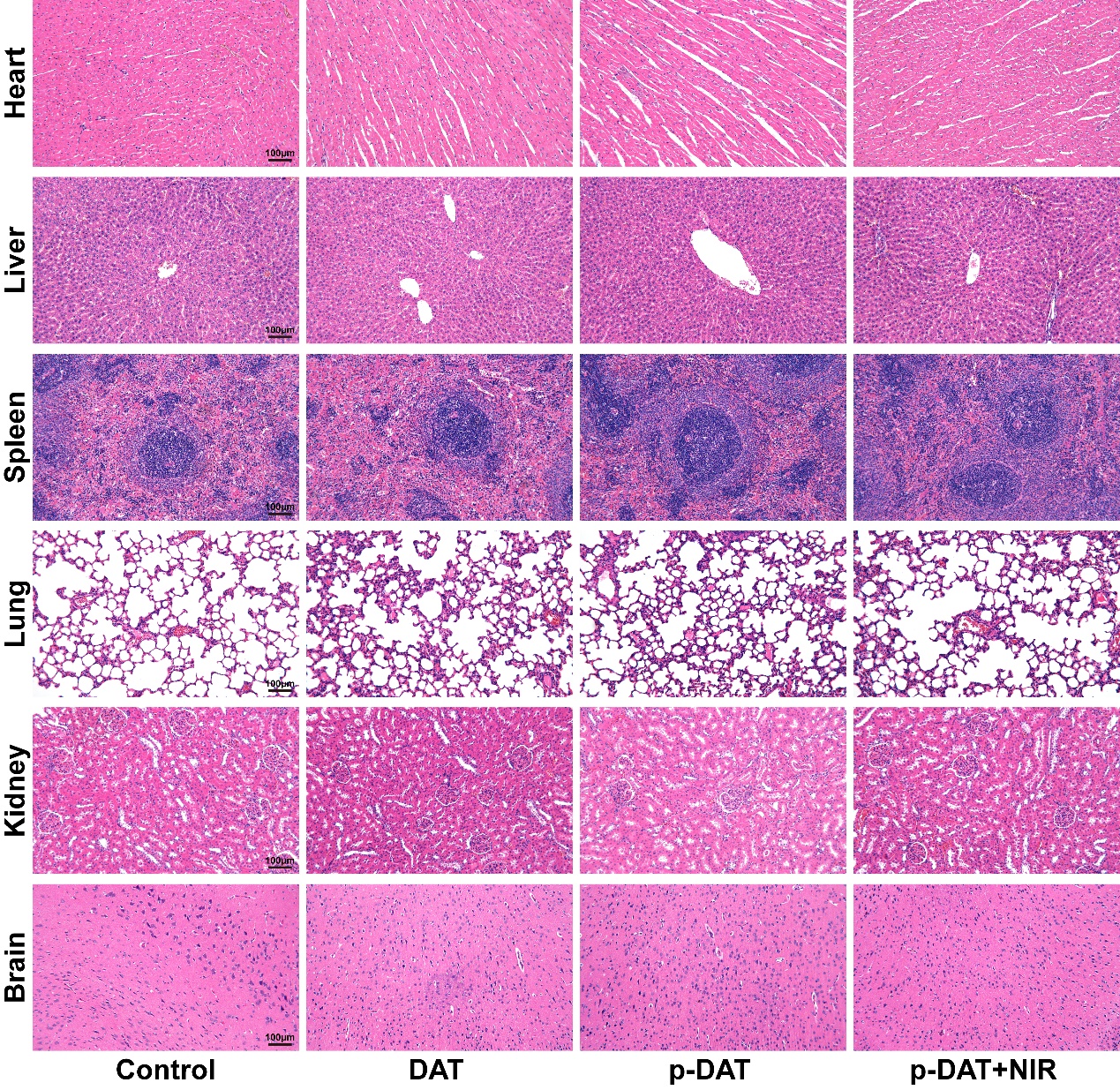


**Figure S22.** Histological images of H & E stained organs (hearts, livers, spleens, lungs and kidneys) harvested from different treated groups (Control, DAT, p-DAT and p-DAT+NIR).
